# Multicenter study on satisfaction, stress and working conditions in nursing in Latin American countries *

**DOI:** 10.1590/1518-8345.7337.4392

**Published:** 2024-11-22

**Authors:** Diana Carolina Tiga-Loza, Anyela Mancilla-Lucumi, Maria Andrea Castro-Bernal, Oscar Javier Vergara-Escobar, Dora Marcela Llanganate-Osorio, Ernesto Gabriel Reimundo Acosta

**Affiliations:** ^1^ Universidad Industrial de Santander, Bucaramanga, Colombia.; ^2^ Universidad Manuela Beltrán, Bogotá, Colombia.; ^3^ Fundación Universitaria del Área Andina, Bogotá, Colombia.; ^4^ Fundación Universitaria Juan N Corpas, Bogotá, Colombia.; ^5^ Universidad Antonio Nariño, Popayán, Colombia.; ^6^ Universidad Nacional de Salta, Salta, Argentina.

**Keywords:** Occupational Stress, Coronavirus, Work Overload, Job Satisfaction, Nursing Research, Multicenter

## Abstract

to assess the job satisfaction level, stressors and working conditions of nursing professionals in some Latin American countries during the COVID-19 pandemic.

this is a cross-sectional study of nursing professionals working in hospital, primary care, educational, governmental, research, commercial and independent institutions. An online survey was carried out to assess satisfaction with the Font-Roja instrument, sociodemographic characteristics, stress factors and working conditions.

a total of 1,215 workers from Argentina, Colombia, Mexico, Ecuador and Panama participated. The overall satisfaction score was 81.5 (SD:11.6). There was less satisfaction in the dimensions of tension, pressure, extrinsic characteristics and monotony. Increased stress at work (84.3%), workload (81.3%), fear of SARS-CoV-2 (67.7%), uncertainty at work (51.2%) and salary reduction (20.4%) were reported. The work factors related to satisfaction were, as follows: job recommendation, mixed working hours, care work, increased stress, increased workload, salary reduction and purchase of protective equipment.

the aspects of job satisfaction most affected during the pandemic were pressure, tension, lack of independence and monotony at work; this satisfaction was reduced due to increased stress and workload, the acquisition of personal protective equipment and a reduction in salary.

## Highlights

**(1)** Job satisfaction in Latin America is perceived as a stress factor.

**(2)** Wage injustice in nursing during the pandemic is highlighted.

**(3)** The pressure and tension perceived by the profession limits their autonomy and independence.

**(4)** Satisfaction is directly related to educational level and the position held.

## Introduction

 During the beginning of 2020, the pandemic caused by the SARS-CoV2 virus became an unprecedented challenge for healthcare workers: the health emergency changed their routines and working conditions, increasing burnout and generating significant work overload ^(^ 1 ^)^ . Nursing professionals have been impacted by the pandemic not only at work, but also on a personal level. This impact can be measured in terms of increased exposure to stressful situations, increased workload, increased likelihood of contagion and increased pressure to face the emergency situation with resilience. All this has led to exodus, job dissatisfaction and even physical and mental health problems ^(^ 2 ^)^ . 

 It is estimated that there are approximately 28 million nursing professionals worldwide and that a third (8.4 million) work in the Region of the Americas. Of this number, 59% are professionals or graduates who provide their services in various areas of activity ^(^ 3 ^)^ . These figures place nursing professionals as the largest workforce during the COVID-19 pandemic. However, the health emergency has put a strain on this workforce caused by exhausting working hours, additional burdens, job desertion and a lack of preparedness on the part of health systems to deal with crisis situations ^(^ 4 ^)^ . 

 As proof of these effects, it has been reported that before the pandemic, only 30% of nurses in the United States of America were willing to look for another job. During the pandemic, however, this figure rose to 60% ^(^ 5 ^)^ . Similarly, in the UK, it was evident that during the pandemic, 60% of nurses were dissatisfied and demoralized with their work, especially when they perceived little support at work after contracting the virus ^(^ 6 ^)^ and a shortage of personal protective equipment (PPE), a situation that led to lower job satisfaction levels ^(^ 7 ^)^ . 

 On the other hand, the COVID-19 pandemic has highlighted job insecurity, not only in unstable employment and contracting conditions, but also in the imposition of a greater workload under the pressure of being fired, a situation that can be considered labor abuse and produce physical and psychological changes in those who suffer it ^(^ 8 ^)^ . 

 For all these reasons, it is necessary to know the working conditions of nursing professionals during this phase of global crisis, especially in the Latin American context, where a greater deterioration in the quality of employment is projected and where nursing is considered to be one of the most vulnerable professions in terms of labor ^(^ 9 ^)^ . The aim of this study was to assess the job satisfaction level, stressors and working conditions of nursing professionals in some Latin American countries during the COVID-19 pandemic ^(^ 10 ^)^ . 

## Method

### Type of study

Cross-sectional, correlational and multicenter study.

### Location and period

 Based on the *Colombia de Red Internacional de Enfermería Basada en la Evidencia* REDIEBE ^(^ 11 ^)^ , Latin American countries in the network were invited to voluntarily participate in the study, establishing Argentina, Colombia, Mexico, Ecuador and Panama as the focus of the study. 

### Population and sample

 Participation was sought from nursing graduates or professionals working in the field as nurses in primary care, hospital, home care, teaching, management, research, business and self-employment, with more than six months’ experience, who wished to participate anonymously and voluntarily. Retirees, students and trainees were excluded from the sample. Sampling was by convenience, taking into account the number of nurses informed by the Pan American Health Organization for each participating country ^(^ 12 ^)^ , with a 95% confidence level and an estimated error of 10% with a 20% loss forecast. The sample size was 805 nurses, but 1,215 nurses responded to the survey, resulting in a sample larger than the estimated size. 

### Study variables and instruments used

An online survey was carried out using a Google Forms form. The survey was completed voluntarily by the participants and included the following sections:

 Socio-demographic data: age, gender, marital status, country and number of dependents were investigated. Socio-economic level was assessed taking into account the classification used by the Social Development Division of the Economic Commission for Latin America and the Caribbean (ECLAC) ^(^ 13 ^)^ and people were allowed to self-identify between the categories of low, medium and high level. 

 Job satisfaction: job satisfaction is understood as the positive or negative judgment that the worker makes about their work situation, from a cognitive perspective that considers the characteristics and attributes that a job should have, as well as from the emotional or affective response in relation to the job as a whole ^(^ 14 ^)^ . To assess satisfaction, we used the Font-Roja questionnaire drawn up by Aranaz ^(^ 15 ^)^ , and obtained the author’s permission to use it. This questionnaire is made up of 9 dimensions (job satisfaction, tension, professional competence, pressure, professional promotion, interpersonal relationship-bosses, interpersonal relationship-colleagues, extrinsic characteristics and monotony) and contains 24 items distributed over the 9 dimensions and evaluated on a 5-point Likert-type scale where 1 means “totally disagree” and 5 “totally agree” for items 6, 7, 12 to 17 and 19, with an inverted score for the other items, obtaining an overall score ranging from 24 to 120 points. The questionnaire also has psychometric characteristics of validity, reliability and internal consistency ^(^ 15 ^-^ 16 ^)^ . 

Stress factors at work: nursing professionals were asked whether they felt increased stress at work due to the pandemic, increased workload, fear of becoming infected with SARS-Cov-2, job instability, reduction in salary and the need to purchase personal protective equipment (PPE) out of their own pocket. Dichotomous answer options (yes/no) were used for these questions.

 Working conditions: the type of institution where the nursing professional worked was investigated so that they could identify with one of the categories, taking into account the place where they had worked for the longest time, being classified *a priori* as follows: 1-Care (hospitals, basic health units or home care), 2-Educational (schools, universities or teaching centers), 3-Public management (government secretariats, ministries, town halls, city councils), 4-Commercial (pharmaceutical industry, product sales), 5-Research (laboratories, national institutes, research centers) and 6-Independent or self-employed (consultant/advisor, contractor). 

The type of employment contract, academic level, years of experience at the time of the survey, type of working hours (day, night or mixed) and weekly working hours were investigated. The questions were linguistically adapted to the labor terms used in each participating country. To do this, employees in the countries were asked to review the survey online and in a peer review, changes were made to the terms that were not easy to understand.

### Data collection and analysis

The survey was publicized through trade unions, work networks, social networks and word of mouth in order to obtain a sample of nursing professionals that matched the target audience. The data was cleaned by excluding those who did not meet the inclusion criteria. The data was described using measures of central tendency and dispersion according to its distribution. Job satisfaction was reported for each item as the mean score together with its respective standard deviation, which covered a range from 0 to 5. In addition, at the end of the report, the overall sum of satisfaction for each of the countries studied was listed.

In addition to the above, the sociodemographic, work and stress characteristics were analyzed. In order to carry out this analysis, in relation to the qualitative variables, the mean job satisfaction score was compared with the sociodemographic and work-related variables using Fisher’s test and Pearson’s chi-square, whereas the t-Student, Anova and trend tests were used for the quantitative variables. Spearman’s correlation was also carried out between job satisfaction and the variables studied. Finally, a linear regression model was used to identify the factors related to job satisfaction. The Stata/17 SE program was used for the analysis.

### Ethical aspects

 This study was approved by the Ethics Committee of the *Universidad Manuela Beltrán* , evaluation term number 20072820, and was carried out taking into account the Nuremberg and Belmont codes and Resolution 8.430 of 1993, section number 2, of the Ministry of Social Protection and Health of Colombia. A virtual informed consent was requested before completing the survey, which is available on the Google form. 

## Results

 Between October 2021 and April 2022, a total of 1,221 nurses participated; two retired nurses and four nursing students were excluded, leaving a final sample of 1,215 participants. In terms of sociodemographic characteristics, the sample was mostly made up of women (79.2%), with a median age of 38 years, of medium socioeconomic status (85.8%), married or living as a couple (51.2%) and undergraduate/graduate students (53.7%) ( Table 1 ). In addition, depending on the country of origin of the interviewees, statistically significant differences were found in each of the variables mentioned: the highest median age was in Ecuador; the lowest proportion of women was in Argentina; the best socio-economic status was in Colombia and the highest proportion of nurses with master’s degrees and doctorates was in Ecuador. 

 In relation to working conditions, it was found that the majority of the sample was made up of nursing professionals working in health institutions such as hospitals, basic health units and home care (73.1%), and that more than half were linked to the companies where they provided their services on an indefinite contract (58.1%). Of this sample, around half worked during the day or in a mixed job (53.6% and 41.7% respectively); the median number of hours worked was 42 and the median number of years of professional experience was 10 years ( Table 1 ). 

 Important differences were also found in working conditions between countries. For example, in Argentina there was a predominance of care nurses with greater contractual stability, while in Colombia there were more nurses who worked during the day with a greater number of hours worked per week. In Ecuador, meanwhile, the participants had more professional experience ( Table 1 ). 

**Table 1 -:** Sociodemographic characteristics and working conditions during the COVID-19 pandemic in nursing professionals from some Latin American countries (n* = 1215). Argentina, Colombia, Mexico, Ecuador and Panama, 2021-2022

**Characteristics**	**Total**	**Argentina**	**Colombia**	**Ecuador**	**Mexico**	**Panama**	***p-* value** ^†^
	n*=1215	n*=512	n*=452	n*=122	n*=66	n*=58	
**Age/Median (IIQ)** ^‡^	38 (14)	40 (15)	36 (12)	**42 (16)**	36 (16)	**35 (17)**	<0.001 ^§^
Female n*(%) ^||^	962 (79.2)	**376 (73.4)**	376 (82.3)	**106 (86.9)**	56 (84.9)	48 (82.8)	0.001 ^¶^
Socioeconomic status n*(%)							
Low	149 (12.3)	**93 (18.2)**	**26 (5.7)**	9 (7.4)	13 (19.7)	8 (13.8)	
Medium	1043 (85.8)	416 (81.3)	413 (90.4)	113 (92.6)	53 (80.3)	48 (82.8)	
High	23 (1.9)	3 (0.6)	18 (3.9)	0 (0)	0 (0)	2 (3.5)	<0.001 ^¶^
Marital status n*(%) ^||^							
Single	464 (38.2)	199 (38.9)	171 (37.4)	41 (33.6)	30 (45.5)	23 (39.7)	
Married or living as a couple	622 (51.2)	249 (48.6)	250 (54.7)	64 (52.5)	28 (42.4)	31 (53.5)	
Divorced or widowed	129 (10.6)	64 (12.5)	36 (7.9)	17 (13.9)	8 (12.1)	4 (6.9)	0.165 ^¶​^
Education level n*(%) ^||^							
Undergraduate-Graduate	652 (53.7)	373 (72.9)	175 (38.3)	24 (19.7)	52 (78.8)	28 (48.3)	
Specialization	268 (22.1)	101 (19.7)	145 (31.7)	4 (3.3)	4 (6.1)	14 (24.1)	
Master’s Degree	269 (22.1)	**36 (7)**	124 (27.1)	**86 (70.5)**	7 (10.6)	16 (27.6)	
Doctorate	26 (2.1)	2 (0.4)	13 (2.8)	8 (6.6)	3 (4.6)	0 (0)	<0.001 ^¶^
Type of institution n*(%) ^||^							
Assistance	888 (73.1)	**466 (91)**	**256 (56)**	56 (45.9)	57 (86.4)	53 (91.4)	
Education, research	227 (18.7)	32 (6.3)	129 (28.2)	61 (50)	3 (4.6)	2 (3.5)	
Public management, administration, commercial	100 (8.2)	14 (2.7)	72 (15.8)	5 (4.1)	6 (9.1)	3 (5.2)	<0.001 ^¶^
Type of contract n*(%) ^||^							
Indefinite term	702 (58.1)	**357 (70.3)**	**197 (43.1)**	63 (52.5)	36 (54.6)	49 (84.5)	
Fixed term	286 (23.7)	87 (17.1)	147 (32.2)	30 (25)	16 (24.2)	6 (10.3)	
Other types of contract	221 (18.3)	64 (12.6)	113 (24.7)	27 (22.5)	14 (21.2)	3 (5.2)	<0.001 ^¶^
Journey n*(%) ^||^							
Daytime work	651 (53.6)	**216 (42.2)**	**301 (65.9)**	76 (62.3)	40 (60.6)	18 (31)	
Mixed shift (day/night)	507 (41.7)	266 (52)	137 (30)	45 (36.9)	19 (28.8)	40 (69)	
Night shift	57 (4.7)	30 (5.9)	19 (4.2)	1 (0.8)	7 (10.6)	0 (0)	<0.001 ^¶^
**Median working hours/week (RI)** ^‡^	42 (10.5)	40 (13)	**48 (6)**	40 (0)	40 (15.5)	40 (12)	0.0001 ^§^
**Mean professional experience (RI)** ^‡^	10 (14)	10 (13)	10 (11)	**17 (17)**	10 (16)	**7.5 (12)**	0.0001 ^§^
Recommends the work n*(%) ^||^							
Yes	42 (10.5)	40 (13)	**48 (6)**	40 (0)	40 (15.5)	40 (12)	
No	1099 (90.59)	444 (86.7)	428 (93.7)	119 (97.5)	58 (87.9)	50 (86.2)	<0.001 ^¶^

Note: The results in bold mark the most extreme adjusted residual values, in relation to the expected values, when the independence test was significant in the *post hoc* analysis

*n Sample size

^†^ *p* *p* -value for 5% significance level

^‡^ IIQ interquartile range

^§^ Pearson’s Chi-square test

^||^ (%) Percentage

^¶^ Kruskal-Wallis test/difference in medians

Table 2 shows the job satisfaction results for each dimension and item according to country. It can be seen that the overall satisfaction means were lower in Argentina and Panama. There is also evidence, at a general level, of lower satisfaction in the dimensions of work-related stress and pressure and work monotony. The dimensions of interpersonal relationships with bosses and colleagues were rated more highly in terms of job satisfaction. 

 When analyzing the data, the least satisfaction was found in items related to worker fatigue, workload, lack of time and independence and monotony. On the other hand, the best ratings were obtained for professional advancement and the relationship between bosses and colleagues. Overall, satisfaction was assessed with a mean score of 81.5 out of 120 points, with a standard deviation (SD) of 11.8 points ( Table 2 ). 

**Table 2 -:** Job satisfaction scores during the COVID-19 pandemic among nursing professionals in five Latin American countries (n* = 1215). Argentina, Colombia, Mexico, Ecuador and Panama, 2021-2022

**Item**		**Total** n*=1215	**Argentina** n*=512	**Colombia** n*=452	Ecuador n*=122	Mexico n*=66	**Panama** n*=58	***p* -value** ^‡^
		**Mean (SD)** ^†^	**Mean (SD)** ^†^	**Mean (SD)** ^†^	**Mean (SD)** ^†^	**Mean (SD)** ^†^	**Mean (SD)** ^†^	
**Dimension 1: Job satisfaction.**		**3.7 (0.7)**	**3.6 (0.7)**	**3.9 (0.7)**	**3.9 (0.7)**	**3.6 (0.6)**	**3.5 (0.7)**	**<0.001**
7	I’m very satisfied with my work.	3.9 (1)	3.8 (1.1)	4 (1)	4.5 (0.7)	4 (1)	3.3 (1.1)	<0.001
10	I have very little interest in the things I do in my job.	3.8 (1.1)	3.7 (1.1)	4.1 (1)	3.7 (1.1)	3.6 (1.2)	3.8 (1.2)	<0.001
11	I have the feeling that what I’m doing isn’t worth it.	3.5 (1.3)	3.3 (1.3)	3.8 (1.3)	3.5 (1.3)	3.4 (1.4)	3.1 (1.4)	<0.001
16	I’m convinced that the position I hold corresponds to my ability and preparation.	3.8 (1.2)	3.7 (1.2)	3.8 (1.1)	4 (1)	3.2 (1.3)	3.7 (1.2)	<0.002
**Dimension 2: Work-related stress**		**2.8 (0.5)**	**2.7 (0.5)**	**2.9 (0.5)**	**2.9 (0.6)**	**3 (0.5)**	**2.6 (0.5)**	**<0.001**
2	I think I have little responsibility in my work.	3.8 (1.1)	3.6 (1.1)	4.2 (1)	3.7 (1.2)	3.6 (1)	3.6 (1.1)	<0.001
3	At the end of a normal working day I feel very tired.	2 (1)	1.9 (1)	4.2 (1)	2.4 (1.1)	2.5 (1.1)	1.7 (1)	<0.001
4	I often find myself thinking about issues related to my work.	2.1 (1)	1.9 (0.9)	2.1 (1)	2.5 (1)	2.6 (1.1)	2.1 (1.1)	<0.001
5	Very rarely have I forced myself to use all my energy and ability to do my job.	2.9 (1.2)	2.8 (1.2)	3.1 (1.3)	3 (1.2)	3 (1.1)	2.7 (1.3)	0.0001
6	Very rarely does my work disturb my mood. my health or my sleep.	3.1 (1.2)	3.1 (1.2)	3 (1.2)	3.2 (1.1)	3.1 (1.2)	3 (1.2)	0.3395
**Dimension 3: Professional competence**		**3.3 (1)**	**3.1 (0.9)**	**3.7 (1)**	**3.3 (1.1)**	**3.3 (0.9)**	**3.2 (1)**	**<0.001**
22	I often have the feeling that I’m not qualified for my job.	3.6 (1.2)	3.5 (1.1)	3.9 (1.2)	3.4 (1.3)	3.5 (1.2)	3.7 (1.2)	<0.001
23	I often feel that I don’t have enough resources to do my job.	3.1 (1.3)	2.7 (1.2)	3.6 (1.2)	3.2 (1.2)	3 (1.2)	2.6 (1.3)	<0.001
24	Competitiveness or the need to measure up to others in my work often causes me tension or stress.	3.3 (1.2)	3 (1.2)	3.5 (1.2)	3.3 (1.2)	3.5 (1)	3.3 (1.2)	<0.001
**Dimension 4: Work pressure**		**2.7 (1)**	**2.6 (0.9)**	**2.7 (1)**	**2.9 (0.9)**	**3 (1)**	**2.4 (0.9)**	**0.0002**
18	I often feel I don’t have time to do my job.	2.5 (1.1)	2.5 (1.1)	2.6 (1.2)	2.6 (1.1)	2.8 (1.1)	2.3 (1)	0.167
20	I think my workload is too much. I can’t cope with what I have to do.	2.9 (1.1)	2.7 (1.1)	2.9 (1.2)	3.2 (1.1)	3.2 (1.2)	2.4 (1.2)	<0.001
**Dimension 5: Professional promotion**		**3.6 (0.7)**	**3.4 (0.7)**	**3.7 (0.7)**	**3.7 (0.7)**	**3.5 (0.6)**	**3.2 (0.7)**	**<0.001**
9	I have few opportunities to learn how to do new things.	3.2 (1.2)	3 (1.2)	3.6 (1.2)	3 (1.2)	3 (1.1)	2.7 (1.2)	<0.001
12	Generally. the recognition I receive for my work is very comforting.	3.4 (1.2)	3.3 (1.2)	3.4 (1.2)	3.8 (1.1)	3.4 (1.3)	2.9 (1.3)	<0.001
17	I have a lot of professional promotion skills.	4.1 (1)	4 (1)	4.2 (1)	4.3 (0.9)	4 (1)	4.1 (1)	0.089
**Dimension 6: Interpersonal relationship with bosses**		**4 (0.8)**	**3.9 (0.8)**	**4.1 (0.7)**	**4.2 (0.7)**	**3.8 (0.8)**	**3.9 (0.8)**	**<0.001**
13	The relationship with my boss is very cordial.	4 (1)	3.9 (1)	4.2 (0.9)	4.3 (0.9)	3.8 (1.1)	3.8 (1)	<0.001
19	I’m sure I know what my bosses expect from my work.	3.9 (0.9)	3.9 (1)	4 (0.9)	4 (0.8)	3.8 (1)	4.1 (0.9)	0.1302
**Dimension 7: Interpersonal relationships with colleagues**		**4.2 (0.8)**	**4.1 (0.9)**	**4.3 (0.7)**	**4.2 (0.8)**	**3.9 (1.1)**	**4.1 (0.7)**	**0.0011**
14	The relationship with my colleagues is very cordial.	4.2 (0.8)	4.1 (0.9)	4.3 (0.7)	4.2 (0.8)	3.9 (1.1)	4.1 (0.7)	0.011
**Dimension 8: Extrinsic characteristics of the job**		**3 (0.8)**	**2.8 (0.8)**	**3.2 (0.9)**	**3.2 (0.7)**	**2.9 (0.6)**	**2.8 (0.7)**	**<0.001**
8	I have little independence when it comes to organizing the work I do. according to my position or professional category.	2.9 (1.1)	2.8 (1.1)	3.1 (1.1)	2.8 (1.2)	2.8 (1)	2.8 (1.2)	0.003
15	The salary I receive is adequate.	3 (1.3)	2.7 (1.2)	3.2 (1.3)	3.7 (1.1)	3 (1.3)	2.9 (1.2)	<0.001
**Dimension 9: Work monotony**		**3.1 (0.9)**	**2.9 (0.8)**	**3.3 (0.9)**	**3 (1)**	**3.1 (0.9)**	**2.8 (1)**	**<0.001**
1	My job is the same every day. it never changes	2.8 (1.2)	2.8 (1.1)	2.9 (1.3)	2.7 (1.3)	2.6 (1.2)	2.4 (1.3)	0.0229
21	The personal problems of my work colleagues often affect me.	3.3 (1.2)	3 (1.1)	3.6 (1.1)	3.3 (1.1)	3.6 (1.2)	3.3 (1.1)	<0.001
**Overall score**		**81.7 (11.8)**	**77.7 (10.1)**	**85.9 (11.7)**	**85.1 (13.4)**	**81.5 (10.6)**	**76.2 (9.8)**	**<0.001**

Note: The scores for each dimension and the overall score are highlighted in bold

*n Sample size

^†^ (SD) Standard deviation

^‡^ p *p* -value for significance level of 5%, obtained by ANOVA test

**Table 3 -:** Work characteristics related to the COVID-19 pandemic in nursing workers in some Latin American countries (n* = 1215). Argentina, Colombia, Mexico, Ecuador and Panama, 2021-2022

**Characteristics**	**Total** n*=1215 n*(%) ^‡^	**Argentina** n*=512 n*(%) ^‡^	**Colombia** n*=452 n*(%) ^‡^	**Ecuador** n*=122 n*(%) ^‡^	**Mexico** n*=66 n*(%) ^‡^	**Panama** n*=58 n*(%) ^‡^	***p* -value** ^†^
Stress at work	1007 (84.3)	**454 (90.3)**	372 (82.7)	83 (70.9)	47 (71.2)	51 (87.9)	<0.001
Fear of being exposed to the virus	796 (67.7)	332 (67.3)	284 (64)	84 (70.6)	**51 (81)**	45 (79)	0.02
Increased workload	972 (81.3)	436 (86.5)	366 (80.6)	74 (62.7)	45 (71.4)	**51 (89.5** )	<0.001
Uncertainty at work	587 (51.2)	239 (50.7)	199 (44.7)	71 (63.4)	41 (65.1)	**37 (66.1** )	<0.001
Salary reduction	219 (20.4)	87 (20)	83 (19.3)	20 (19.2)	16 (30.2)	13 (25)	0.367
Switching to teleworking	431 (35.6)	109 (21.3)	**240 (53.1)**	**68 (55.7)**	10 (15.2)	4 (6.9)	<0.001
Buying PPE out of your own pocket	645 (54.8)	254 (50.2)	206 (47.4)	86 (76.1)	**53 (80.3)**	**46 (79.3)**	<0.001

*n Sample size

^†^ *p* *p* -value for significance level of 5%, obtained by Fisher’s Exact test

^‡^ (%) = Percentage

In relation to stress factors at work during the COVID-19 pandemic, the majority of respondents reported stress (84.3%) and increased workload (81.3%); on the other hand, only 20.4% suffered a salary reduction and 35% reported switching to teleworking. When comparing countries, important differences were found: Argentina reported a higher proportion of stress at work (90.3%; p<0.001), whereas the majority of Mexican professionals expressed fear due to exposure to the COVID-19 virus (81%; p=0.02); for their part, nurses in Panama perceived more overload (89.5%; p<0.001) and uncertainty at work (66.1%; p<0.001); finally, Colombian and Ecuadorian professionals were the ones who most faced the change to teleworking (53.1% and 55.7% respectively; p<0.001).

Finally, a linear regression model was carried out, finding socioeconomic and work factors related to the job satisfaction level of nursing professionals, namely: socioeconomic level, marital status, type of institution where they worked at the time of the survey, recommending or not recommending work, perceived stress, salary reduction, purchase of PPE out of pocket and increased working hours due to the pandemic.

**Figure 1 -:**
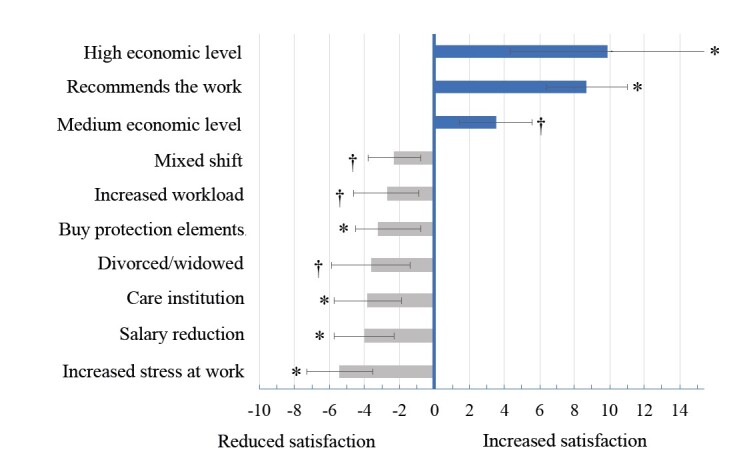
Factors related to job satisfaction scores during the COVID-19 pandemic in nurses from some Latin American countries: Argentina, Colombia, Mexico, Ecuador and Panama, 2021-2022

Note: The gray bars indicate the decrease (negative values) in the total satisfaction index and the blue bars the increase in the total job satisfaction index (positive values) estimated by the β coefficient and obtained by linear regression model. The 95% confidence intervals are represented by the lines above the bars; Regression with a sample of 1,034 participants, r2=0.22; * *p* -value <0.001 for 5% significance level; ^†^ *p* -value <0.01 for 5% significance level

 Consequently, having a medium socioeconomic level increases satisfaction by a mean of 3.5 points (95% CI: 1.4; 5.6), whereas being at a high level increases satisfaction by 9.9 points (95% CI: 4.3;15) when compared to nurses with a low economic level (p < 0.001). Similarly, professionals who recommend their work score a mean of 8.7 points (95% CI: 6.4;11) higher than those who do not. On the other hand, job satisfaction levels were reduced on mean by 2.3 points (95% CI: -3.8;-0.8) in those who alternate between day and night shifts and by 3.8 points (95% CI: -5.7;-1.9) in those who work in health institutions, comparing these data with teaching or research institutions. Similarly, as a result of the pandemic, satisfaction fell by 5.4 points (95% CI: -7.3;-3.5) among those who had more stress at work, 4 points (95% CI: -5.7;-2.3) among those who saw their salary reduced, 3.2 points (95% CI: -4.5;-0.8) among those who had to buy PPE out of their own pocket and 2.7 points (95% CI: -4.6;-0.9) among those who reported an increase in workload ( Figure 1 ). 

## Discussion

 In this study, a moderate job satisfaction level was observed among nursing professionals, with differences between the participating countries. A higher satisfaction index was also found for Colombia compared to that reported in 2014 by 105 nursing assistants in the city of Bogotá, Colombia (69.98 SD: 5.67) using the same Font-Roja instrument ^(^ 17 ^)^ . On the other hand, in Mexico in 2012, a higher mean job satisfaction was reported (101; SD:10) based on the results of 594 nurses from four health institutions ^(^ 18 ^)^ . On the other hand, in Córdoba, Argentina, it was found that of 333 surveys carried out in public hospitals, the highest dissatisfaction rate was related to professional growth in the institution and the amount of remuneration ^(^ 19 ^)^ . 

The items with the greatest dissatisfaction (both intrinsic and extrinsic) were those that in some way assess how workers perceive their possibilities for growth, both within the institution and economically. Thus, a significant proportion of the professionals surveyed face their daily tasks with the perception that they do not receive an adequate salary or have the possibility of promotion in the place where they work.

These disparate results can be explained by the context of the pandemic, as well as the type of institutions to which the participants are affiliated. An example of this is that our study found that working in the health sector was associated with lower satisfaction compared to other areas.

 Similarly, in this study women took part in greater numbers, although no differences were found in satisfaction by gender. Contrary to this, it was observed that nurses have had lower job satisfaction compared to men ^(^ 20 ^)^ and higher job stress ^(^ 21 ^)^ . The training and employment processes in nursing have been predominantly female, since the act of caring is socially linked to women ^(^ 22 ^)^ , and according to the Pan American Health Organization (PAHO) report for the Region of the Americas, approximately 87% of nursing staff are women ^(^ 10 ^)^ . This means that women are the majority in the care workforce and consequently have a greater care burden ^(^ 23 ^)^ . 

 Another interesting finding was the relationship between socioeconomic status and job satisfaction. This phenomenon has already been described, considering salary and its relationship with satisfaction ^(^ 24 ^)^ , finding that the better the economic level, the better the perception of satisfaction. In addition to the socio-economic level obtained through salary, it is important to take other aspects into account when assessing job satisfaction, such as long working hours and the need for time and rest ^(^ 25 ^)^ . In addition, it was observed that nurses perceive a pay gap when comparing their salaries with those earned by other professions with less responsibility or workload, a pay gap that may be determined by gender discrimination, causing professional dissatisfaction in a profession with a predominance of women ^(^ 26 ^)^ . 

 It is noteworthy that, in the health care sector, the pandemic has had a greater impact on work, due to long working hours, lack or loss of staff, risk of infection and limitations in personal protective equipment ^(^ 2 ^)^ . For example, in Israel, nurses’ job satisfaction was assessed during the pandemic, and was lower among those who worked in hospitals, tested positive for COVID-19 or had poor personal protective equipment ^(^ 7 ^)^ . In addition, there is greater dissatisfaction when nurses are unable to provide the necessary care to patients, when they lack support and supervision, when there is a shortage of staff or when they feel unable to take a break and have to work overtime ^(^ 6 ^)^ . Although teaching nurses reported higher mean satisfaction than care nurses, this does not mean that they did not face multiple work challenges, such as the need to adapt abruptly to remote educational technologies. 

 Another important aspect to be analyzed is workload, which is considered to be the stressor most frequently and negatively related to job satisfaction and perceived quality of care ^(^ 27 ^)^ . For example, those who worked in Intensive Care Units had greater exposure to stressors ^(^ 28 ^)^ and increased frequency of symptoms of anxiety, depression and post-traumatic stress ^(^ 29 ^)^ . 

 Employment also plays a key role in worker well-being. This study found that only 58.1% of the participating nursing workers had an indefinite contract. Although working conditions prior to the pandemic indicated the need to reduce informality, wages below the legal minimum or the lack of social security, it is possible that, with the pandemic, these phenomena have worsened ^(^ 9 ^)^ . This is why stable contracts should be favored, as they have been shown to have a positive effect on attitudes towards communication, emotional intelligence and empathy ^(^ 30 ^)^ , as well as favoring workers’ health in aspects such as stress levels, quality of sleep, burnout, staff turnover rates and service optimization ^(^ 31 ^-^ 33 ^)^ . 

 In addition to the above, the pandemic has also caused changes in remuneration for around 30% of the nurses who took part in the study. It was considered that the perception of a low salary was also a stressful factor for nursing staff during the pandemic, especially when the workload, fatigue and risk of infection increased ^(^ 34 ^)^ . Still, in Lebanon, 38.3% of nurses who worked during the pandemic did so because they needed a salary for their families, even if they wanted to resign ^(^ 35 ^)^ . 

 On the other hand, the COVID-19 pandemic has forced a change in work dynamics, with lower job satisfaction being reported by day and night shift workers, as well as an increase in workload, greater stress at work and job uncertainty, with important differences between the participating countries. In addition, satisfaction was lower for items related to workload, tiredness and lack of time and independence. Similarly, in Mexico, nurses who worked more than 12 hours a day during the pandemic suffered greater stress ^(^ 36 ^)^ , and in Uruguay 52.4% of respondents worked up to 12 hours, 96.1% saw their work pace accelerate and 87% felt an increase in workload when the pandemic began, compared to what they normally had ^(^ 37 ^)^ . Similarly, in Peru, 38.8% of nurses reported moderate stress during the pandemic ^(^ 38 ^)^ and in Ecuador, more than 90% of doctors and nurses reported professional burnout ^(^ 39 ^)^ . The fear of being exposed to the virus was another of the participants’ concerns at work (65.4%). This fear arises mainly in the provision of direct care, which denotes vulnerability to a scenario of greater working hours and job instability ^(^ 40 ^)^ . 

All these findings show the need for Latin American countries to establish policies that guarantee fairer working conditions, without disparities between the different areas in which nurses work, as is the case in Colombia, where a median working week of 48 hours was reported, much higher than the other participating countries.

 In addition, this study provides evidence of how some sociodemographic and labor aspects affect the perception of job satisfaction among nursing professionals, especially in countries where labor fragility persists due to hiring conditions, overload and organizational climate, as well as the lack of preparation of health systems ^(^ 41 ^)^ . 

Although it was difficult to get the participation of nursing professionals in the different Latin American countries due to the dynamics of the pandemic and the limitations of time available, the nursing associations were a facilitator for data collection. It is hoped that future studies will assess the perception of satisfaction and its relationship with the quality of life or health of the worker, or in situations such as multiple jobs or simultaneous performance of duties, aspects not assessed in this study.

## Conclusion

The job satisfaction of nurses in some Latin American countries during the pandemic has decreased due to the perception of pressure and tension, lack of independence and monotony. In addition, nursing professionals were more satisfied with their work when they had a higher socioeconomic status and when their marital status was single, married or living as a couple.

In addition, in terms of working conditions, satisfaction was lower among those who worked in healthcare environments, on mixed shifts (day and night) or did not recommend their work. Faced with the implications the pandemic has had on work, satisfaction decreased for those who perceived an increase in workload, greater stress and a reduction in salary. The above confirms that the COVID-19 pandemic has indeed had a negative impact on job insecurity and therefore on nurses’ job satisfaction in some Latin American countries.

Protecting nurses’ job security is crucial. International and national standards that can be applied alongside labor policies are needed to improve nurses’ employment conditions and work-life balance. Collective bargaining through participation in unions and networks can be a way to demand fairer employment and working conditions.
