# Supplementary material for: Multicenter study on satisfaction, stress and working conditions in nursing in Latin American countries
Source: Rev Lat Am Enfermagem. 2024 Nov 22;32:e4392. doi: 10.1590/1518-8345.7337.4392 (PMC11654028; doi:10.1590/1518-8345.7337.4392)
Supplement: Translation

# Translation: Estudio multicéntrico sobre la satisfacción, estrés y condiciones laborales en enfermería en países latinoamericanos *

**DOI:** 10.1590/1518-8345.7337.4391

**Published:** 2024-11-15

**Authors:** Diana Carolina Tiga-Loza, Anyela Mancilla-Lucumi, Maria Andrea Castro-Bernal, Oscar Javier Vergara-Escobar, Dora Marcela Llanganate-Osorio, Ernesto Gabriel Reimundo Acosta

**Affiliations:** ^1^ Universidad Industrial de Santander, Bucaramanga, Colombia.; ^2^ Universidad Manuela Beltrán, Bogotá, Colombia.; ^3^ Fundación Universitaria del Área Andina, Bogotá, Colombia.; ^4^ Fundación Universitaria Juan N Corpas, Bogotá, Colombia.; ^5^ Universidad Antonio Nariño, Popayán, Colombia.; ^6^ Universidad Nacional de Salta, Salta, Argentina.

**Keywords:** Estrés Ocupacional, Coronavirus, Sobrecarga de Trabajo, Satisfacción Laboral, Investigación en Enfermería, Multicéntrico

## Abstract

evaluar el nivel de satisfacción laboral, los estresores y las condiciones laborales de profesionales de enfermería en algunos países latinoamericanos durante la pandemia por COVID-19.

estudio transversal con profesionales de enfermería activos laboralmente en instituciones hospitalarias, de atención primaria, educativas, gubernamentales, de investigación, comerciales e independientes. Se realizó una encuesta por internet que evaluó la satisfacción con el instrumento Font-Roja, las características sociodemográficas, los factores de estrés y las condiciones de trabajo.

participaron 1.215 trabajadores de Argentina, Colombia, México, Ecuador y Panamá. El puntaje global de satisfacción fue 81,5 (DE:11,6). Hubo menor satisfacción en las dimensiones de tensión, presión, características extrínsecas y monotonía. Se reportó un incrementó del estrés laboral (84,3%), carga laboral (81,3%), miedo al SARS-CoV-2 (67,7%), incertidumbre laboral (51,2%) y reducción salarial (20,4%). Los factores laborales relacionados con la satisfacción fueron: recomendar el trabajo, jornada mixta, trabajo asistencial, incremento del estrés, aumento de la carga, reducción salarial y compra de elementos de protección.

los aspectos de la satisfacción laboral más afectados durante la pandemia fueron la presión, tensión, falta de independencia y monotonía laboral; esta satisfacción se redujo ante el aumento del estrés y la carga laboral, compra de elementos de protección personal y reducción salarial.

## Puntos destacados

**(1)** Se evidencia injusticia salarial en Enfermería durante la pandemia

**(2)** La presión y tensión percibida por la profesión limitan su autonomía e independencia.

**(3)** La satisfacción está directamente relacionada con el nivel educativo y el cargo desempeñado.

## Introducción

 Durante el inicio del 2020, la pandemia provocada por el virus SARS-CoV-2 se convirtió en un desafío sin precedentes para los trabajadores de la salud: la emergencia sanitaria modificó sus rutinas y condiciones de trabajo, incrementando el agotamiento y generando una sobrecarga laboral importante ^(^ 1 ^)^ . Los profesionales en enfermería han sufrido el impacto de la pandemia no solo a nivel laboral, sino también a nivel personal. Este impacto puede medirse en términos de una exposición mayor a situaciones estresantes, un aumento en la carga de trabajo, una mayor probabilidad de contagio y un incremento de la presión por afrontar con resiliencia la situación de emergencia. Todo lo anterior ha conducido al éxodo, a la insatisfacción laboral e incluso a los problemas en la salud física y mental ^(^ 2 ^)^ . 

 Se estima que existen aproximadamente 28 millones de profesionales de enfermería en todo el mundo, y que una tercera parte (8,4 millones) trabaja en la Región de las Américas. De este número, 59% corresponde a profesionales o licenciados que prestan sus servicios en diferentes campos de acción ^(^ 3 ^)^ . Estas cifras posicionan a los profesionales de la enfermería como la mayor fuerza laboral durante la pandemia por COVID-19; sin embargo, la emergencia sanitaria produjo en esta fuerza un desgaste causado por las jornadas extenuantes de trabajo, las cargas adicionales, la deserción laboral y la falta de preparación de los sistemas de salud para enfrentar situaciones de crisis ^(^ 4 ^)^ . 

 Como prueba de estos efectos, se ha reportado que, antes de la pandemia, solo el 30% de los enfermeros en Estados Unidos de América estaban dispuestos a buscar otro trabajo. Durante la pandemia, sin embargo, esta cifra ascendió al 60% ^(^ 5 ^)^ . De modo semejante, en Reino Unido se evidenció que, en época de pandemia, 60% de los enfermeros estaban insatisfechos y desmoralizados con su trabajo, en especial cuando percibieron poco apoyo laboral tras contagiarse el virus ^(^ 6 ^)^ y escasez de elementos de protección personal (EPP), situación que generó menores niveles de satisfacción laboral ^(^ 7 ^)^ . 

 Por otro lado, la pandemia por COVID-19 evidenció la precariedad laboral, no solo en las condiciones inestables de vinculación y contratación, sino también en la imposición de mayor carga laboral ante una presión de despido, situación que puede considerarse como maltrato laboral y producir alteraciones físicas y psicológicas en quien lo padece ^(^ 8 ^)^ . 

 Por todo lo anterior, es necesario que se conozcan las condiciones laborales de los profesionales de enfermería durante esta etapa de crisis mundial, en especial en el contexto latinoamericano, donde se ha proyectado un mayor deterioro en la calidad del empleo y donde se ha considerado que enfermería es una de las profesiones vulnerables laboralmente ^(^ 9 ^)^ . El objetivo del presente estudio fue evaluar el nivel de satisfacción laboral, los estresores y las condiciones laborales de los profesionales de enfermería en algunos países latinoamericanos durante la pandemia por COVID-19 ^(^ 10 ^)^ . 

## Método

### Tipo de estudio

Estudio transversal correlacional, multicéntrico.

### Lugar y periodo

 Desde el nodo Colombia de Red Internacional de Enfermería Basada en la Evidencia REDIEBE ^(^ 11 ^)^ , se invitó a participar de manera voluntaria en el estudio a los representantes de los países latinoamericanos de la red, estableciéndose como centros del estudio los países de Argentina, Colombia, México, Ecuador y Panamá. 

### Población y muestra

 Se buscó la participación de licenciados o profesionales de enfermería activos laboralmente como enfermeros en roles de atención primaria, hospitalaria, domiciliaria, docencia, gerencia, investigación, comerciales y de trabajo independiente, con más de seis meses de experiencia, y que desearan participar de forma anónima y voluntaria. De la muestra se excluyeron jubilados, estudiantes o pasantes. El muestreo fue por conveniencia, teniendo en cuenta el número de enfermeros reportados por la Organización Panamericana de la Salud para cada país participante ^(^ 12 ^)^ , un nivel de confianza del 95% y un error estimado del 10% con previsión del 20% en pérdidas. El tamaño de muestra fue de 805 enfermeros, sin embargo, 1.215 enfermeros contestaron la encuesta, obteniendo una muestra superior al tamaño estimado. 

### Variables de estudio e instrumentos utilizados

 Se realizó una encuesta por internet a través de un formulario de *Google Forms* . La encuesta fue autocompletada por los participantes de forma voluntaria y contaba con los siguientes apartados: 

 Datos sociodemográficos: edad, el sexo, el estado civil, el país, el número de personas a cargo de los profesionales. El nivel socioeconómico fue evaluado teniendo en cuenta la clasificación usada por la División de Desarrollo Social de la Comisión Económica para América Latina y el Caribe (CEPAL) ^(^ 13 ^)^ y se permitió que las personas se autoidentificaran entre las categorías de nivel bajo, medio y alto. 

 Satisfacción laboral: la satisfacción laboral es entendida como el juicio positivo o negativo que el trabajador hace sobre su situación de trabajo, desde la óptica cognitiva que contempla las características y atributos que debe tener un trabajo, así como desde la respuesta emocional o afectiva hacia el trabajo como un todo ^(^ 14 ^)^ . Para evaluar la satisfacción, se utilizó el cuestionario Font-Roja diseñado por Aranaz ^(^ 15 ^)^ , obteniéndose permiso por el autor para su uso. Este cuestionario está constituido por 9 dimensiones (satisfacción por el trabajo, tensión, competencia profesional, presión, promoción profesional, relación interpersonal-jefes, relación interpersonal-compañeros, características extrínsecas y monotonía), contiene 24 ítems distribuidos en las 9 dimensiones y valorados con una escala tipo Likert de 5 puntos en que 1 significa “muy en desacuerdo” y 5 “muy de acuerdo” para los ítems 6, 7, 12-17 y 19, con una puntuación invertida para los demás ítems, obteniéndose un puntaje global que va de 24 a 120 puntos. El cuestionario, además, cuenta con características psicométricas de validez, confiabilidad y consistencia interna ^(^ 15 ^-^ 16 ^)^ . 

Cada uno de los ítems es valorado mediante una escala Likert, cuyos valores oscilan entre 1 (totalmente en desacuerdo) y 5 (totalmente de acuerdo). Existen ítems que a mayor puntuación indican mayor satisfacción (7, 8, 9, 10, 11, 12, 13, 14, 15, 16, 17 y 19), y otros que a mayor puntuación nos indica una menor satisfacción (1, 2, 3, 4, 5, 6, 18, 20, 21, 22, 23 y 24).

Factores de estrés laboral: se preguntó si los profesionales de enfermería por ocasión de la pandemia habían sentido un incremento en el estrés laboral, mayor carga de trabajo, miedo a infectarse por SARS-CoV-2, inestabilidad laboral, reducción del salario y necesidad de comprar elementos de protección personal (EPP) de su bolsillo. Se emplearon opciones de respuesta dicotómicas (sí/no) para estas preguntas.

 Condiciones laborales: se indagó sobre el tipo de institución donde trabajaba el profesional de enfermería para que se identificaran con alguna de las categorías, teniendo en cuenta el lugar donde mayor tiempo de dedicación laboral emplearon, clasificándose *a priori* de la siguiente manera: 1-Asistencial (hospitales, centros de atención primaria o cuidado domiciliario), 2-Educativa (escuelas, universidades o centros de enseñanza), 3-Gestión pública (secretarías de gobierno, ministerios, alcaldías, ayuntamientos), 4-Comerciales (industria farmacéutica, venta de productos), 5-Investigación (laboratorios, institutos nacionales, centros de investigación) y 6-Independiente o negocio propio (consultor/asesor, contratista). 

 Además, se indagó sobre el tipo de contrato laboral, el nivel académico, los años de experiencia al momento de la encuesta, el tipo de jornada (diurna, nocturna o mixta) y las horas de trabajo por semana. Se realizó una adaptación lingüística de las preguntas en cuanto a los términos laborales usados en cada país participante, para esto, se solicitó a los colaboradores de los países la revisión de la encuesta *online* y en una revisión por pares se realizaron los cambios de los términos que no fueran de fácil comprensión. 

### Recolección y análisis de datos

La encuesta fue difundida por agremiaciones, redes de trabajo, redes sociales y voz a voz para obtener una muestra de profesionales de enfermería que coincidiera con la población objetivo. Se realizó una limpieza de datos excluyendo a quienes no cumplieran los criterios de inclusión. Los datos se describieron usando medidas de tendencia central y dispersión según su distribución. La satisfacción laboral se reportó, por cada ítem, como el puntaje medio junto a su respectiva desviación estándar, la cual abarcó un intervalo de 0 a 5. Además, al final del reporte, se relacionó la sumatoria global de satisfacción para cada uno de los cinco países estudiados.

 A lo anterior, se suma el análisis de las características sociodemográficas, laborales y de estrés. Para llevar a cabo este análisis, en lo que respecta a las variables cualitativas, se comparó la media del puntaje de satisfacción laboral con las variables sociodemográficas y laborales mediante la prueba de Fisher y el Chi-cuadrado de Pearson; para las variables cuantitativas, se utilizaron las pruebas T-Student, ANOVA y de tendencia. Además, se realizó una correlación de Spearman entre la satisfacción laboral y las variables estudiadas. Finalmente, para identificar los factores que se relacionaron con la satisfacción laboral, se utilizó un modelo de regresión lineal. Para el análisis se usó el programa *Stata/1* 7 *SE.*

### Consideraciones éticas

 Este estudio fue aprobado por el Comité de Ética de la Universidad Manuela Beltrán en acta de evaluación número 20072820, y realizado en consideración de los códigos de Núremberg y Belmont y de la resolución 8430 de 1993, apartado número 2, del Ministerio de Protección Social y Salud de Colombia. Se solicitó un consentimiento informado virtual previo al llenado de la encuesta que estada disponible en el formulario de *Google* . 

## Resultados

 Entre octubre de 2021 y abril de 2022 participaron 1.221 profesionales de enfermería, se excluyeron dos jubilados y cuatro estudiantes de enfermería, quedando una muestra final de 1.215 participantes. Con respecto a las características sociodemográficas, la muestra estuvo compuesta, en su mayoría, por mujeres (79,2%) con una mediana de edad de 38 años, de nivel socioeconómico medio (85,8%), casadas o conviviendo con pareja (51,2%) y con pregrado/licenciatura (53,7%) ( Tabla 1 ). Además, en función del país de origen de los encuestados, se encontraron diferencias estadísticamente significativas en cada una de las variables antes mencionadas: la mediana más alta de edad se presenta en Ecuador; la menor proporción de mujeres, en Argentina; la mejor condición socioeconómica se halla en Colombia y la proporción más alta de enfermeros con nivel de maestría y doctorado, en Ecuador. 

 Frente a las condiciones laborales, se encontró que la muestra estuvo conformada, en su mayoría, por profesionales de enfermería que trabajaban en instituciones asistenciales como hospitales, centros de atención primaria y cuidado domiciliario (73,1%), y que más de la mitad estaban vinculados a las empresas donde prestaban sus servicios con un contrato a término indefinido (58,1%). De esta muestra, alrededor de la mitad debía cumplir una jornada de trabajo diurna o mixta (53,6% y 41,7% respectivamente); la mediana de horas trabajadas fue de 42 y la de experiencia laboral fue de 10 años ( Tabla 1 ). 

 También se encontraron diferencias importantes en las condiciones laborales entre los países; por ejemplo, en Argentina predominó la participación de enfermeros asistenciales con mayor estabilidad contractual, mientras que, en Colombia, hubo más enfermeros que trabajaban en jornada diurna con la mayor cantidad de horas trabajadas por semana. En Ecuador, por su parte, los participantes contaban con mayor experiencia laboral ( Tabla 1 ). 

**Tabla 1 -:** Características sociodemográficas y condiciones laborales durante la pandemia por COVID-19 en los profesionales de enfermería en algunos países de Latinoamérica (n* = 1.215). Argentina, Colombia, México, Ecuador y Panamá, 2021-2022

**Características**	**Total**	**Argentina**	**Colombia**	**Ecuador**	**México**	**Panamá**	**Valor *p*** ^†^
	n*=1215	n*=512	n*=452	n*=122	n*=66	n*=58	
**Edad/Mediana (RI)** ^‡^	38 (14)	40 (15)	36 (12)	**42 (16)**	36 (16)	**35 (17)**	<0,001 ^§^
Sexo femenino n*(%) ^||^	962 (79,2)	**376 (73,4)**	376 (82,3)	**106 (86,9)**	56 (84,9)	48 (82,8)	0,001 ^¶^
Nivel socioeconómico n*(%)							
Bajo	149 (12,3)	**93 (18,2)**	**26 (5,7)**	9 (7,4)	13 (19,7)	8 (13,8)	
Medio	1043 (85,8)	416 (81,3)	413 (90,4)	113 (92,6)	53 (80,3)	48 (82,8)	
Alto	23 (1,9)	3 (0,6)	18 (3,9)	0 (0)	0 (0)	2 (3,5)	<0,001 ^¶^
Estado civil, n*(%) ^||^							
Soltero(a)	464 (38,2)	199 (38,9)	171 (37,4)	41 (33,6)	30 (45,5)	23 (39,7)	
Casado(a) o conviviendo en pareja	622 (51,2)	249 (48,6)	250 (54,7)	64 (52,5)	28 (42,4)	31 (53,5)	
Divorciado(a) o viudo(a)	129 (10,6)	64 (12,5)	36 (7,9)	17 (13,9)	8 (12,1)	4 (6,9)	0,165 ^¶^
Nivel educativo, n*(%) ^||^							
Pregrado-licenciatura	652 (53,7)	373 (72,9)	175 (38,3)	24 (19,7)	52 (78,8)	28 (48,3)	
Especialización	268 (22,1)	101 (19,7)	145 (31,7)	4 (3,3)	4 (6,1)	14 (24,1)	
Maestría	269 (22,1)	**36 (7)**	124 (27,1)	**86 (70,5)**	7 (10,6)	16 (27,6)	
Doctorado	26 (2,1)	2 (0,4)	13 (2,8)	8 (6,6)	3 (4,6)	0 (0)	<0,001 ^¶^
Tipo de institución, n*(%) ^||^							
Asistencial	888 (73,1)	**466 (91)**	**256 (56)**	56 (45,9)	57 (86,4)	53 (91,4)	
Educativa, investigación	227 (18,7)	32 (6,3)	129 (28,2)	61 (50)	3 (4,6)	2 (3,5)	
Gestión pública, administración, comercial	100 (8,2)	14 (2,7)	72 (15,8)	5 (4,1)	6 (9,1)	3 (5,2)	<0,001 ^¶^
Tipo de contrato n*(%) ^||^							
A término indefinido	702 (58,1)	**357 (70,3)**	**197 (43,1)**	63 (52,5)	36 (54,6)	49 (84,5)	
A término fijo	286 (23,7)	87 (17,1)	147 (32,2)	30 (25)	16 (24,2)	6 (10,3)	
Otro tipo de contrato	221 (18,3)	64 (12,6)	113 (24,7)	27 (22,5)	14 (21,2)	3 (5,2)	<0,001 ^¶^
Jornada n*(%) ^||^							
Jornada diurna	651 (53,6)	**216 (42,2)**	**301 (65,9)**	76 (62,3)	40 (60,6)	18 (31)	
Jornada mixta (diurna/nocturna)	507 (41,7)	266 (52)	137 (30)	45 (36,9)	19 (28,8)	40 (69)	
Jornada nocturna	57 (4,7)	30 (5,9)	19 (4,2)	1 (0,8)	7 (10,6)	0 (0)	<0,001 ^¶^
**Horas de trabajo/semana mediana (RI)** ^‡^	42 (10,5)	40 (13)	**48 (6)**	40 (0)	40 (15,5)	40 (12)	0,0001 ^§^
**Experiencia laboral, mediana (RI)** ^‡^	10 (14)	10 (13)	10 (11)	**17 (17)**	10 (16)	**7,5 (12)**	0,0001 ^§^
Recomienda el trabajo n*(%) ^||^							
Sí	42 (10,5)	40 (13)	**48 (6)**	40 (0)	40 (15,5)	40 (12)	
No	1099 (90,59)	444 (86,7)	428 (93,7)	119 (97,5)	58 (87,9)	50 (86,2)	<0,001 ^¶^

Nota: Los valores en negrita tuvieron residuales ajustados más extremos, con respecto a los valores esperados, cuando la prueba de independencia fue significativa en análisis *post hoc*

*n Tamaño de muestra

^†^p Valor *p* para un nivel de significancia del 5%

^‡^ RI Rango intercuartílico

§ Prueba Chi-cuadrado de Pearson

^||^ (%) Porcentaje

¶ Prueba de Kruskal-Wallis/diferencia de medianas

 En la Tabla 2 , se presentan los resultados de satisfacción laboral para cada dimensión e ítem según el país. Se observa allí que las medias de satisfacción global fueron más bajas en Argentina y Panamá. También se evidencia, a nivel general, una menor satisfacción en las dimensiones de tensión y presión relacionadas con el trabajo y monotonía laboral. Las dimensiones de relaciones interpersonales con los jefes y compañeros fueron calificadas con una mayor satisfacción laboral. 

 Al analizar los datos, se observó una menor satisfacción en ítems relacionados con la fatiga/cansancio del trabajador, la carga laboral, la falta de tiempo e independencia y la monotonía. Por el contrario, la mejor valoración se obtuvo en las capacidades de promoción profesional y en la relación entre jefes y compañeros. De forma global, la satisfacción fue valorada con un puntaje medio de 81,5 sobre 120 puntos, con una desviación estándar (DE) de 11,8 puntos ( Tabla 2 ). 

**Tabla 2 -:** Puntajes de satisfacción laboral durante la pandemia por COVID-19 en profesionales de enfermería en cinco países de Latinoamérica (n* = 1.215). Argentina, Colombia, México, Ecuador y Panamá, 2021-2022

**Ítem**		**Total** n*=1215	**Argentina** n*=512	**Colombia** n*=452	Ecuador n*=122	**México** n*=66	**Panamá** n*=58	**Valor *p*** ^‡^
		**Media (DE)** ^†^	**Media (DE)** ^†^	**Media (DE)** ^†^	**Media (DE)** ^†^	**Media (DE)** ^†^	**Media (DE)** ^†^	
**Dimensión 1: Satisfacción por el trabajo.**		**3,7 (0,7)**	**3,6 (0,7)**	**3,9 (0,7)**	**3,9 (0,7)**	**3,6 (0,6)**	**3,5 (0,7)**	**<0,001**
7	En mi trabajo me encuentro muy satisfecho.	3,9 (1)	3,8 (1,1)	4 (1)	4,5 (0,7)	4 (1)	3,3 (1,1)	<0,001
10	Tengo muy poco interés por las cosas que realizo en mi trabajo.	3,8 (1,1)	3,7 (1,1)	4,1 (1)	3,7 (1,1)	3,6 (1,2)	3,8 (1,2)	<0,001
11	Tengo la sensación de que lo que estoy haciendo no vale la pena.	3,5 (1,3)	3,3 (1,3)	3,8 (1,3)	3,5 (1,3)	3,4 (1,4)	3,1 (1,4)	<0,001
16	Estoy convencido de que el puesto de trabajo que ocupo me corresponde por mi capacidad y preparación.	3,8 (1,2)	3,7 (1,2)	3,8 (1,1)	4 (1)	3,2 (1,3)	3,7 (1,2)	<0,002
**Dimensión 2: Tensión relacionada con el trabajo**		**2,8 (0,5)**	**2,7 (0,5)**	**2,9 (0,5)**	**2,9 (0,6)**	**3 (0,5)**	**2,6 (0,5)**	**<0,001**
2	Creo que tengo poca responsabilidad en mi trabajo.	3,8 (1,1)	3,6 (1,1)	4,2 (1)	3,7 (1,2)	3,6 (1)	3,6 (1,1)	<0,001
3	Al final de la jornada de un día de trabajo corriente y normal me encuentro muy cansado.	2 (1)	1,9 (1)	2 (1)	2,4 (1,1)	2,5 (1,1)	1,7 (1)	<0,001
4	Con bastante frecuencia me he sorprendido pensando en cuestiones relacionadas con mi trabajo	2,1 (1)	1,9 (0,9)	2,1 (1)	2,5 (1)	2,6 (1,1)	2,1 (1,1)	<0,001
5	Muy pocas veces me he obligado a emplear a tope toda mi energía y capacidad para realizar mi trabajo.	2,9 (1,2)	2,8 (1,2)	3,1 (1,3)	3 (1,2)	3 (1,1)	2,7 (1,3)	0,0001
6	Muy pocas veces mi trabajo perturba mi estado de ánimo, mi salud o mis horas de sueño.	3,1 (1,2)	3,1 (1,2)	3 (1,2)	3,2 (1,1)	3,1 (1,2)	3 (1,2)	0,3395
**Dimensión 3: Competencia profesional**		**3,3 (1)**	**3,1 (0,9)**	**3,7 (1)**	**3,3 (1,1)**	**3,3 (0,9)**	**3,2 (1)**	**<0,001**
22	Con frecuencia tengo la sensación de no estar capacitado para mi trabajo.	3,6 (1,2)	3,5 (1,1)	3,9 (1,2)	3,4 (1,3)	3,5 (1,2)	3,7 (1,2)	<0.001
23	Con frecuencia siento no tener recursos suficientes para realizar mi trabajo.	3,1 (1,3)	2,7 (1,2)	3,6 (1,2)	3,2 (1,2)	3 (1,2)	2,6 (1,3)	<0.001
24	Con frecuencia la competitividad o el estar a la altura de los demás en mi puesto de trabajo me causa tensión o estrés.	3,3 (1,2)	3 (1,2)	3,5 (1,2)	3,3 (1,2)	3,5 (1)	3,3 (1,2)	<0.001
**Dimensión 4: Presión del trabajo**		**2,7 (1)**	**2,6 (0,9)**	**2,7 (1)**	**2,9 (0,9)**	**3 (1)**	**2,4 (0,9)**	**0,0002**
18	Con frecuencia tengo la sensación de que me falta tiempo para realizar mi trabajo.	2,5 (1,1)	2,5 (1,1)	2,6 (1,2)	2,6 (1,1)	2,8 (1,1)	2,3 (1)	0.167
20	Creo que mi trabajo es excesivo, no doy abasto con lo que tengo que hacer.	2,9 (1,1)	2,7 (1,1)	2,9 (1,2)	3,2 (1,1)	3,2 (1,2)	2,4 (1,2)	<0.001
**Dimensión 5: Promoción profesional**		**3,6 (0,7)**	**3,4 (0,7)**	**3,7 (0,7)**	**3,7 (0,7)**	**3,5 (0,6)**	**3,2 (0,7)**	**<0,001**
9	Tengo pocas oportunidades para aprender a hacer cosas nuevas.	3,2 (1,2)	3 (1,2)	3,6 (1,2)	3 (1,2)	3 (1,1)	2,7 (1,2)	<0.001
12	Generalmente el reconocimiento que obtengo en mi trabajo es muy reconfortante.	3,4 (1,2)	3,3 (1,2)	3,4 (1,2)	3,8 (1,1)	3,4 (1,3)	2,9 (1,3)	<0.001
17	Tengo muchas capacidades de promoción profesional.	4,1 (1)	4 (1)	4,2 (1)	4,3 (0,9)	4 (1)	4,1 (1)	0.089
**Dimensión 6: Relación interpersonal con los jefes**		**4 (0,8)**	**3,9 (0,8)**	**4,1 (0,7)**	**4,2 (0,7)**	**3,8 (0,8)**	**3,9 (0,8)**	**<0,001**
13	La relación con mi jefe es muy cordial.	4 (1)	3,9 (1)	4,2 (0,9)	4,3 (0,9)	3,8 (1,1)	3,8 (1)	<0.001
19	Estoy seguro de conocer lo que se espera de mi trabajo, por parte de mis jefes.	3,9 (0,9)	3,9 (1)	4 (0,9)	4 (0,8)	3,8 (1)	4,1 (0,9)	0.1302
**Dimensión 7: Relación interpersonal con los compañeros**		**4,2 (0,8)**	**4,1 (0,9)**	**4,3 (0,7)**	**4,2 (0,8)**	**3,9 (1,1)**	**4,1 (0,7)**	**0,0011**
14	La relación con mis compañeros es muy cordial.	4,2 (0,8)	4,1 (0,9)	4,3 (0,7)	4,2 (0,8)	3,9 (1,1)	4,1 (0,7)	0.011
**Dimensión 8: Características extrínsecas del trabajo**		**3 (0,8)**	**2,8 (0,8)**	**3,2 (0,9)**	**3,2 (0,7)**	**2,9 (0,6)**	**2,8 (0,7)**	**<0,001**
8	Para organizar el trabajo que realizo, según mi puesto o categoría profesional, tengo poca independencia.	2,9 (1,1)	2,8 (1,1)	3,1 (1,1)	2,8 (1,2)	2,8 (1)	2,8 (1,2)	0.003
15	El sueldo que recibo es adecuado.	3 (1,3)	2,7 (1,2)	3,2 (1,3)	3,7 (1,1)	3 (1,3)	2,9 (1,2)	<0.001
**Dimensión 9: Monotonía laboral**		**3,1 (0,9)**	**2,9 (0,8)**	**3,3 (0,9)**	**3 (1)**	**3,1 (0,9)**	**2,8 (1)**	**<0,001**
1	Mi trabajo es el mismo todos los días, nunca cambia.	2,8 (1,2)	2,8 (1,1)	2,9 (1,3)	2,7 (1,3)	2,6 (1,2)	2,4 (1,3)	0.0229
21	Los problemas personales de mis compañeros de trabajo, habitualmente me suelen afectar.	3,3 (1,2)	3 (1,1)	3,6 (1,1)	3,3 (1,1)	3,6 (1,2)	3,3 (1,1)	<0.001
**Puntaje global**		**81,7 (11,8)**	**77,7 (10,1)**	**85,9 (11,7)**	**85,1 (13,4)**	**81,5 (10,6)**	**76,2 (9,8)**	**<0,001**

Nota: En negrita se resaltan los puntajes para cada dimensión y el puntaje global

*n Tamaño de muestra

^†^ (DE) Desviación estándar

^‡^ p Valor p para un nivel de significancia del 5%, obtenido mediante prueba de ANOVA

**Tabla 3 -:** Características laborales relacionadas con la pandemia por COVID-19 en el personal de enfermería en algunos países de Latinoamérica (n ^*^ = 1.215). Argentina, Colombia, México, Ecuador y Panamá, 2021-2022

**Características**	**Total** n*=1215 n*(%) ^‡^	**Argentina** n*=512 n*(%) ^‡^	**Colombia** n*=452 n*(%) ^‡^	**Ecuador** n*=122 n*(%) ^‡^	**México** n*=66 n*(%) ^‡^	**Panamá** n*=58 n*(%) ^‡^	**Valor *p*** ^†^
Estrés laboral	1007 (84,3)	**454 (90,3)**	372 (82,7)	83 (70,9)	47 (71,2)	51 (87,9)	<0,001
Miedo por tener que exponerse al virus	796 (67,7)	332 (67,3)	284 (64)	84 (70,6)	**51 (81)**	45 (79)	0,02
Aumento de la carga laboral	972 (81,3)	436 (86,5)	366 (80,6)	74 (62,7)	45 (71,4)	**51 (89,5** )	<0,001
Incertidumbre laboral	587 (51,2)	239 (50,7)	199 (44,7)	71 (63,4)	41 (65,1)	**37 (66,1** )	<0,001
Reducción en el salario	219 (20,4)	87 (20)	83 (19,3)	20 (19,2)	16 (30,2)	13 (25)	0,367
Cambios a teletrabajo	431 (35,6)	109 (21,3)	**240 (53,1** )	**68 (55,7)**	10 (15,2)	4 (6,9)	<0,001
Comprar EPP de su bolsillo	645 (54,8)	254 (50,2)	206 (47,4)	86 (76,1)	**53 (80,3)**	**46 (79,3)**	<0,001

*n Tamaño de muestra

^†^ p Valor p para un nivel de significancia del 5%, obtenido mediante la prueba Exacta de Fisher

^‡^ (%) Porcentaje

En cuanto a los factores de estrés laboral durante la pandemia por COVID-19, la mayoría de los encuestados reportó estrés (84,3%) y aumento de la carga laboral (81,3%); por el contrario, sólo 20,4% sufrieron reducción del salario y 35% manifestaron cambios a teletrabajo. Al comparar entre países, se encontraron diferencias importantes: en Argentina se reportó mayor proporción de estrés laboral (90,3%; p<0,001), mientras que la mayoría de profesionales mexicanos manifestaron miedo por exposición al virus del COVID-19 (81%; p=0,02); por su parte, los enfermeros de Panamá percibieron más carga (89,5%; p<0,001) e incertidumbre laboral (66,1%; p<0,001); los profesionales colombianos y ecuatorianos fueron quienes más enfrentaron un cambio al teletrabajo (53,1% y 55,7% respectivamente; p<0,001).

Por último, se realizó un modelo de regresión lineal, encontrándose factores socioeconómicos y laborales relacionados con el nivel de satisfacción laboral de los profesionales de enfermería, a saber: nivel socioeconómico, estado civil, tipo de institución donde trabajaban en el momento de la encuesta, recomendar o no el trabajo, estrés percibido, reducción del salario, compra de EPP del propio bolsillo y aumento en la carga laboral por la pandemia.

 Tener un nivel socioeconómico medio aumentó en promedio 3,5 puntos (IC95%:1,4;5,6) la satisfacción, mientras que estar en un nivel alto aumentó la satisfacción en 9,9 puntos (IC 95%:4,3;15,5) en comparación con enfermeros de nivel económico bajo (p<0,001). De igual forma, los profesionales que recomendaron su trabajo presentaron una media de 8,7 puntos (IC95%:6.4;11) por encima de aquellos que no lo recomiendaron. Por el contrario, los niveles de satisfacción laboral se redujeron en promedio en 2,3 puntos (IC95%: -3.8;-0.8) en quienes alternaron entre jornadas diurnas y nocturnas y en 3,8 puntos (IC95%:-5.7;-1.9) en quienes trabajaron en instituciones asistenciales, comparando este dato con las instituciones educativas o dedicadas a la investigación. Asimismo, por ocasión de la pandemia, la satisfacción bajó puntos 5,4 puntos (IC95%:-7.3;-3.5) en quienes tuvieron mayor estrés laboral, 4 puntos (IC95%:-5.7;-2.3) en quienes vieron reducido su salario, 3,2 puntos (IC95%:-4.5;-0.8) en quienes tuvieron que comprar EPP de su bolsillo y 2,7 puntos (IC95%:-4.6;-0.9) en los que refirieron aumento en la carga laboral ( Figura 1 ). 

**Figura 1 -:** Factores relacionados con los puntajes de satisfacción laboral durante la pandemia por COVID-19 en enfermeros en algunos países de Latinoamérica. Argentina, Colombia, México, Ecuador y Panamá, 2021-2022

Nota: Las barras grises indican la reducción (valores negativos) del puntaje total de satisfacción y las azules el incremento del puntaje total de satisfacción laboral (valores positivos) estimados mediante el coeficiente β y obtenido por un modelo de regresión lineal. Los intervalos de confianza del 95% están representados por las líneas sobre las barras; Regresión con una muestra de 1.034 participantes, r2=0,22; *Valor p <0,001 para un nivel de significancia del 5%; ^†^ Valor p<0,01 para un nivel de significancia del 5%

## Discusión

 En este estudio se observó un nivel moderado en la satisfacción laboral de los profesionales de enfermería, con diferencias entre los países participantes. También se encontró un puntaje más alto de satisfacción para Colombia comparado con el reportado en 2014 por 105 enfermeros asistenciales de la ciudad de Bogotá-Colombia, (69,98 DE:5,67) a través del mismo instrumento Font-Roja ^(^ 17 ^)^ . Por el contrario, en México en el 2012 se reportó una media de satisfacción laboral más alta (101; DE:10) a partir de los hallazgos en 594 enfermeras/os de cuatro instituciones de salud ^(^ 18 ^)^ . Por otro lado, en Córdoba, Argentina, se evidenció que, de 333 encuestas realizadas en hospitales públicos, el mayor índice de insatisfacción se relaciona con el crecimiento profesional en la institución y con el valor de la remuneración ^(^ 19 ^)^ . 

Los ítems en los que se registró mayor falta de satisfacción (tanto intrínseca como extrínseca) fueron aquellos que de alguna manera evaluaran cómo los trabajadores percibieron sus posibilidades de crecimiento, tanto en la institución como en lo económico. Así, una importante proporción de profesionales encuestados afrontaba su tarea diaria con la percepción de que no recibían un salario adecuado ni tenían posibilidades de ascender en el lugar en el que presta servicio.

Estos resultados disímiles pueden explicarse por el contexto de la pandemia, así como por el tipo de instituciones a las que están vinculados los participantes. Un ejemplo de esto es que en nuestro estudio se encontró que trabajar en el ámbito asistencial se asoció a una satisfacción más baja en comparación con otras áreas laborales.

 Asimismo, en este estudio las mujeres participaron en mayor proporción, aunque no se encontraron diferencias en la satisfacción por sexo. Contrario a esto, se ha observado que las mujeres enfermeras han tenido una menor satisfacción laboral en comparación con los hombres ^(^ 20 ^)^ y mayor estrés laboral ^(^ 21 ^)^ . Los procesos de formación y de vinculación laboral en enfermería han sido de predominio femenino, pues el acto de cuidar se vincula socialmente a las mujeres ^(^ 22 ^)^ , además de acuerdo con el informe de la Organización Panamericana de la Salud (OPS) para la Región de las Américas, aproximadamente 87% del personal de enfermería son mujeres ^(^ 10 ^)^ . Esto significa que las mujeres son la mayoría en la fuerza laboral del cuidado y en consecuencia tienen una mayor carga a causa del cuidado ^(^ 23 ^)^ . 

 Otro hallazgo de interés fue la relación entre el nivel socioeconómico y la satisfacción laboral, este fenómeno ha sido previamente descrito en cuanto al salario y su relación con la satisfacción ^(^ 24 ^)^ , encontrándose que, a mejor nivel económico, mejor percepción de la satisfacción. Además del nivel socioeconómico obtenido a través del salario, es importante tener en cuenta otros aspectos al valorar la satisfacción por el trabajo, como las largas jornadas laborales y las necesidades de tiempo y descanso ^(^ 25 ^)^ . Además, se ha observado que las enfermeras perciben injusticia salarial, al ser comparados sus haberes con lo que devengan otras profesiones de menor responsabilidad o carga laboral, brecha salarial que puede estar determinada por discriminación de género, provocando insatisfacción laboral en una profesión con predominio del sexo femenino ^(^ 26 ^)^ . 

 Se resalta que, en ámbitos asistenciales, la pandemia causó una mayor afectación laboralmente, por los turnos extendidos, la falta o baja de personal, el riesgo de infección y la limitación en los equipos de protección personal ^(^ 2 ^)^ . Por ejemplo, en Israel se evaluó la satisfacción laboral de los enfermeros durante la pandemia, siendo más baja en quienes que trabajaban en hospitales, dieron positivo en la prueba de COVID-19 o tuvieron precariedad de elementos de protección personal ^(^ 7 ^)^ . Además, hay mayor insatisfacción cuando los enfermeros no logran brindar el cuidado necesario a los pacientes, por no tener apoyo y supervisión, cuando falta personal, o sienten incapacidad para tomar un descanso y deben trabajar horas extras ^(^ 6 ^)^ . Si bien los enfermeros docentes reportaron una media de satisfacción mayor a las asistenciales, no significa que no hayan tenido que afrontar múltiples retos laborales, como la necesidad de adaptación abrupta a las tecnologías educativas remotas. 

 Otro aspecto importante para analizar es la carga de trabajo, considerada como el factor estresante que más frecuentemente y de manera negativa se relaciona con la satisfacción laboral y la calidad percibida de la atención ^(^ 27 ^)^ . Por ejemplo, quienes trabajaron en Unidades de Cuidados Intensivos, tuvieron mayor exposición a factores estresantes ^(^ 28 ^)^ y un incremento en la frecuencia de síntomas de ansiedad, depresión y estrés postraumático ^(^ 29 ^)^ . 

 La vinculación laboral también juega un papel fundamental en el bienestar del trabajador. En el presente estudio se encontró que sólo 58,1% del personal de enfermería participante, tenían un contrato a término indefinido. Si bien las condiciones laborales antes de la pandemia indicaban la necesidad de disminuir la informalidad, el salario inferior al mínimo legal o la falta de seguridad social; es posible que, con la pandemia, estos fenómenos se hayan agudizado ^(^ 9 ^)^ . Por tal razón, se debe propender por contratos estables, puesto que han mostrado un efecto positivo en las actitudes hacia la comunicación, la inteligencia emocional y la empatía ^(^ 30 ^)^ , además de favorecer la salud del trabajador en aspectos como el nivel de estrés, la calidad del sueño, el *burnout* o en las tasas de rotación de personal y la optimización del cuidado ^(^ 31 ^-^ 33 ^)^

 Aunado a lo anterior, la pandemia también provocó modificaciones en la remuneración salarial en cerca de 30% de los enfermeros que respondieron la encuesta. Se ha considerado que la percepción de un bajo salario también fue un factor estresante del personal de enfermería durante la pandemia, más aún cuando se incrementó la carga de trabajo, la fatiga y el riesgo de infección ^(^ 34 ^)^ . Aun así, en el Líbano, 38,3% de los enfermeros que trabajaban durante la pandemia lo hacían porque necesitan un salario para sus familias, aunque quisieran darse de baja ^(^ 35 ^)^ . 

 Por otro lado, la pandemia por COVID-19 forzó a que las dinámicas laborales cambiaran, reportándose menor satisfacción tanto en quienes tuvieron turnos de trabajo de día como de noche, así como un aumento de la carga laboral, mayor estrés en el trabajo e incertidumbre laboral, con diferencias importantes entre los países participantes. Además, la satisfacción fue más baja en los ítems relacionados con la carga laboral, la fatiga y la falta de tiempo e independencia. De forma similar, en México, los enfermeros que trabajaban más de 12 horas por día durante la pandemia sufrieron de mayor estrés ^(^ 36 ^)^ , y en Uruguay 52,4% de los encuestados trabajaron hasta 12 horas, 96,1% vieron acelerado su ritmo de trabajo y 87% sintieron mayor carga laboral a la que usualmente les correspondían una vez entrada la pandemia ^(^ 37 ^)^ ; así mismo, en Perú 38,8% de los enfermeros manifestaron estrés moderado durante la pandemia ^(^ 38 ^)^ y en Ecuador más del 90% de los médicos y enfermeros manifestaron desgaste por el trabajo ^(^ 39 ^)^ . El miedo por tener que exponerse al virus, fue otra de las preocupaciones laborales de los participantes (65,4%). Este miedo principalmente surge al momento de brindar los cuidados directos, lo que denota vulnerabilidad ante un escenario de mayor carga e incertidumbre laboral ^(^ 40 ^)^ . 

Todos estos hallazgos dan muestra de la necesidad de que los países en Latinoamérica establezcan políticas que garanticen condiciones laborales más justas, sin brechas entre los diferentes ámbitos de desempeño de los enfermeros, como en el caso de Colombia, donde se reportó una mediana de 48 horas de trabajo por semana, muy superior a los demás países participantes.

 Además, en este estudio se da evidencia de como algunos aspectos sociodemográficos y laborales afectan la satisfacción laboral percibida de los profesionales de enfermería, particularmente en países donde persiste la fragilidad laboral por las condiciones de contratación, la sobrecarga y el clima organizacional, además de la falta de preparación de los sistemas de salud ^(^ 41 ^)^ . 

Aunque haya sido difícil lograr la participación de profesionales de enfermería en diferentes países de Latinoamérica dada las dinámicas de la pandemia y las limitaciones de disponibilidad de tiempo, las agremiaciones de enfermería fueron una vía facilitadora para la recolección de datos. Se espera que, en futuros estudios, se evalué la percepción de la satisfacción y su relación con la calidad de vida o la salud del trabajador, o ante situaciones como el trabajo múltiple o el desempeño simultáneo de roles, aspectos no evaluados en este estudio.

## Conclusión

La satisfacción laboral de enfermería en algunos países de Latinoamérica durante la pandemia se vio disminuida por la presión y la tensión percibida, la falta de independencia y la monotonía; además, los profesionales de enfermería estuvieron más satisfechos con su trabajo cuando tenían un mayor nivel socioeconómico y cuando su estado civil era soltero, casado o convivían en pareja.

Además, en cuanto a condiciones laborales, la satisfacción fue más baja en quienes trabajaban en ámbitos asistenciales, en jornadas mixtas (diurna y nocturna) o no recomendaban su trabajo. Frente a las implicaciones que tuvo la pandemia sobre el trabajo, la satisfacción se redujo para quienes percibieron un aumento en la carga laboral, mayor estrés y reducción del salario. Lo anterior denota que, en efecto, la pandemia por COVID-19 tuvo un impacto negativo sobre la precariedad laboral y por ende en la satisfacción con el trabajo de los enfermeros en algunos países de Latinoamérica.

La protección de la seguridad laboral de los enfermeros es primordial. Se necesitan normas internacionales y nacionales que puedan ser aplicadas junto a políticas laborales para mejorar las condiciones de empleo de los enfermeros y el equilibrio entre la vida personal y laboral. La negociación colectiva a través de la participación en agremiaciones y redes puede ser una vía de exigencia de empleo y condiciones laborales más justas.

# Translation: Estudo multicêntrico sobre satisfação, estresse e condições de trabalho na Enfermagem em países latino-americanos *

**DOI:** 10.1590/1518-8345.7337.4393

**Published:** 2024-11-15

**Authors:** Diana Carolina Tiga-Loza, Anyela Mancilla-Lucumi, Maria Andrea Castro-Bernal, Oscar Javier Vergara-Escobar, Dora Marcela Llanganate-Osorio, Ernesto Gabriel Reimundo Acosta

**Affiliations:** ^1^ Universidad Industrial de Santander, Bucaramanga, Colômbia.; ^2^ Universidad Manuela Beltrán, Bogotá, Colômbia.; ^3^ Fundación Universitaria del Área Andina, Bogotá, Colômbia.; ^4^ Fundación Universitaria Juan N Corpas, Bogotá, Colômbia.; ^5^ Universidad Antonio Nariño, Popayán, Colômbia.; ^6^ Universidad Nacional de Salta, Salta, Argentina.

**Keywords:** Estresse Ocupacional, Coronavírus, Sobrecarga de Trabalho, Satisfação no Trabalho, Pesquisa em Enfermagem, Multicêntrico

## Abstract

avaliar o nível de satisfação profissional, os estressores e as condições de trabalho dos profissionais de enfermagem em alguns países da América Latina durante a pandemia de COVID-19.

estudo transversal realizado com profissionais de enfermagem atuantes em instituições hospitalares, de atenção primária, educacionais, governamentais, de pesquisa, comerciais e independentes. Foi realizada uma pesquisa *online* que avaliou a satisfação com o instrumento *Font-Roja* , as características sociodemográficas, os fatores de estresse e as condições de trabalho.

participaram 1.215 trabalhadores da Argentina, Colômbia, México, Equador e Panamá. O escore geral de satisfação foi 81,5 (DP:11,6). Houve menor satisfação nas dimensões tensão, pressão, características extrínsecas e monotonia. Foi relatado aumento no estresse no trabalho (84,3%), carga de trabalho (81,3%), medo do SARSCoV-2 (67,7%), incerteza no trabalho (51,2%) e redução salarial (20,4%). Os fatores laborais relacionados à satisfação foram: recomendação do emprego, jornada mista, trabalho assistencial, aumento do estresse, aumento da carga horária, redução salarial e compra de elementos de proteção.

os aspectos da satisfação no trabalho mais afetados durante a pandemia foram pressão, tensão, falta de independência e monotonia no trabalho; esta satisfação foi reduzida devido ao aumento do estresse e da carga de trabalho, aquisição de elementos de proteção individual e redução salarial.

## Destaques

**(1)** A satisfação no trabalho na América Latina é percebida como um fator de estresse.

**(2)** É evidenciada a injustiça salarial na Enfermagem durante a pandemia.

**(3)** A pressão e a tensão percebidas pela profissão limitam a sua autonomia e independência.

**(4)** A satisfação está diretamente relacionada ao nível educacional e ao cargo ocupado.

## Introdução

 Durante o início de 2020, a pandemia causada pelo vírus SARS-CoV2 tornou-se um desafio sem precedentes para os trabalhadores da saúde: a emergência sanitária modificou suas rotinas e condições de trabalho, aumentando o esgotamento e gerando uma sobrecarga de trabalho significativa ^(^ 1 ^)^ . Os profissionais de enfermagem têm sofrido o impacto da pandemia não só no nível laboral, mas também no nível pessoal. Este impacto pode ser medido em termos de maior exposição a situações estressantes, aumento da carga de trabalho, maior probabilidade de contágio e aumento da pressão para enfrentar a situação de emergência com resiliência. Estas condições levaram ao êxodo, à insatisfação no trabalho e até mesmo a problemas de saúde física e mental ^(^ 2 ^)^ . 

 Estima-se que existam aproximadamente 28 milhões de profissionais de enfermagem em todo o mundo e que um terço (8,4 milhões) trabalhe na Região das Américas. Desse número, 59% correspondem a profissionais ou graduados que prestam seus serviços em diversas áreas de atuação ^(^ 3 ^)^ . Estes números posicionam os profissionais de enfermagem como a maior força de trabalho durante a pandemia da COVID-19. Contudo, a emergência sanitária produziu desgastes nesta força de trabalho causados por jornadas exaustivas de trabalho, cargas adicionais, deserção de emprego e falta de preparo dos sistemas de saúde para enfrentar situações de crise ^(^ 4 ^)^ . 

 Como prova destes efeitos, foi relatado que, antes da pandemia, apenas 30% dos enfermeiros nos Estados Unidos da América estavam dispostos a procurar outro emprego. Durante a pandemia, porém, esse número subiu para 60% ^(^ 5 ^)^ . Da mesma forma, no Reino Unido foi evidente que, durante a pandemia, 60% dos enfermeiros estavam insatisfeitos e desmoralizados com o seu trabalho, especialmente quando perceberam pouco apoio laboral após contraírem o vírus ^(^ 6 ^)^ e escassez de equipamentos de proteção individual (EPIs), situação que gerou níveis mais baixos de satisfação no trabalho ^(^ 7 ^)^ . 

 Por outro lado, a pandemia de COVID-19 evidenciou a insegurança laboral, não só nas condições instáveis de emprego e contratação, mas também na imposição de uma maior carga de trabalho sob a pressão de ser demitido, situação que pode ser considerada abuso laboral e produzir alterações físicas e psicológicas em quem a sofre ^(^ 8 ^)^ . 

 Por tudo isso, é necessário conhecer as condições de trabalho dos profissionais de enfermagem durante esta fase de crise global, especialmente no contexto latino-americano, onde se projeta uma maior deterioração na qualidade do emprego e se considera que a enfermagem é uma das profissões laboralmente vulneráveis ^(^ 9 ^)^ . O objetivo do presente estudo foi avaliar o nível de satisfação no trabalho, os estressores e as condições de trabalho dos profissionais de enfermagem em alguns países da América Latina durante a pandemia de COVID-19 ^(^ 10 ^)^ . 

## Método

### Tipo de estudo

Estudo transversal, correlacional e multicêntrico.

### Local e período

 A partir do eixo Colômbia da *Red Internacional de Enfermería Basada en la Evidencia* REDIEBE ^(^ 11 ^)^ , países latino-americanos da rede foram convidados a participar voluntariamente do estudo, estabelecendo os países Argentina, Colômbia, México, Equador e Panamá como focos do estudo. 

### População e amostra

 Buscou-se a participação de graduados em enfermagem ou de profissionais atuantes na área como enfermeiros com desempenho nos âmbitos da atenção primária, hospitalar, domiciliar, docência, gerência, pesquisa, comerciais e de trabalho independente, com mais de seis meses de experiência, e que desejassem participar de forma anônima e voluntária. Foram excluídos da amostra os aposentados, estudantes ou estagiários. A amostragem foi por conveniência, levando em consideração o número de enfermeiros informado pela Organização Pan-Americana da Saúde para cada país participante ^(^ 12 ^)^ , com nível de confiança de 95% e erro estimado de 10% com previsão de 20% de perdas. O tamanho da amostra foi de 805 enfermeiros, porém, 1.215 enfermeiros responderam à pesquisa, obtendo-se uma amostra maior que o tamanho estimado. 

### Variáveis do estudo e instrumentos utilizados

 Foi realizada uma pesquisa *online* por meio de um formulário *do Google Forms* . A pesquisa foi autopreenchida pelos participantes voluntariamente e incluiu as seguintes seções: 

 Dados sociodemográficos: idade, sexo, estado civil, país e número de pessoas a cargo dos profissionais. O nível socioeconômico foi avaliado levando-se em consideração a classificação utilizada pela Divisão de Desenvolvimento Social da Comissão Econômica para a América Latina e o Caribe (CEPAL) ^(^ 13 ^)^ e as pessoas foram autorizadas a se autoidentificar entre as categorias de nível baixo, médio e alto. 

 Satisfação no trabalho: a satisfação no trabalho é entendida como o julgamento positivo ou negativo que o trabalhador faz sobre sua situação de trabalho, desde uma perspectiva cognitiva que contempla as características e atributos que um trabalho deve ter, bem como a partir da resposta emocional ou afetiva em relação ao trabalho como um todo ^(^ 14 ^)^ . Para avaliar a satisfação foi utilizado o questionário *Font-Roja* elaborado por Aranaz ^(^ 15 ^)^ , obtendo autorização do autor para sua utilização. Este questionário é composto por 9 dimensões (satisfação no trabalho, tensão, competência profissional, pressão, promoção profissional, relacionamento interpessoal-chefes, relacionamento interpessoal-colegas, características extrínsecas e monotonia), com 24 itens distribuídos nas 9 dimensões e avaliados em uma escala tipo Likert de 5 pontos na qual 1 significa “discordo totalmente” e 5 “concordo totalmente” para os itens 6, 7, 12 a 17 e 19, utilizando pontuação invertida para os demais itens, obtendo uma pontuação global que varia de 24 a 120 pontos. O questionário também possui características psicométricas de validade, confiabilidade e consistência interna ^(^ 15 ^-^ 16 ^)^ . 

Fatores de estresse no trabalho: foi questionado se os profissionais de enfermagem devido à pandemia sentiram aumento do estresse no trabalho, maior carga horária, medo de se infectar pelo SARS-Cov-2, instabilidade no emprego, redução de salário e necessidade de aquisição de equipamentos de proteção individual (EPIs) pagando do próprio bolso. Foram utilizadas opções de resposta dicotômicas (sim/não) para essas questões.

 Condições de trabalho: investigou-se o tipo de instituição onde o profissional de enfermagem trabalhava para que ele pudesse se identificar com uma das categorias, levando em consideração o local onde trabalharam por mais tempo, sendo classificado *a priori* da seguinte forma: 1-Assistencial (hospitais, unidades básicas de saúde ou atenção domiciliar), 2-Educacional (escolas, universidades ou centros de ensino), 3-Gestão pública (secretarias de governo, ministérios, prefeituras, câmaras municipais), 4-Comercial (indústria farmacêutica, venda de produtos), 5-Pesquisa (laboratórios, institutos nacionais, centros de pesquisa) e 6-Independente ou atividade por conta própria (consultor/assessor, contratista). 

 Foram investigados o tipo de contrato de trabalho, o nível acadêmico, os anos de experiência no momento da pesquisa, o tipo de jornada laboral (diurna, noturna ou mista) e a jornada de trabalho semanal. Foi realizada uma adaptação linguística das perguntas em relação aos termos trabalhistas utilizados em cada país participante. Para isso, os colaboradores dos países foram solicitados a revisar a pesquisa *online* e em uma revisão por pares foram feitas as alterações dos termos que não fossem de fácil compreensão. 

### Coleta e análise de dados

A pesquisa foi divulgada por meio de sindicatos, redes de trabalho, redes sociais e boca a boca para obter uma amostra de profissionais de enfermagem que coincidisse com o público-alvo. O refinamento dos dados foi realizada excluindo aqueles que não atendiam aos critérios de inclusão. Os dados foram descritos por meio de medidas de tendência central e dispersão de acordo com sua distribuição. A satisfação no trabalho foi relatada, para cada item, como a pontuação média juntamente com seu respectivo desvio-padrão, que abrangeu um intervalo de 0 a 5. Além disso, ao final do relatório, foi relacionada a soma geral de satisfação para cada um dos países estudados.

 Ao exposto, acrescenta-se a análise das características sociodemográficas, laborais e de estresse. Para realizar esta análise, no que diz respeito às variáveis qualitativas, comparou-se a média do escore de satisfação no trabalho com as variáveis sociodemográficas e laborais por meio do teste de Fisher e do qui-quadrado de Pearson, para as variáveis quantitativas foram utilizados os testes t-Student, Anova e tendência. Adicionalmente, foi realizada correlação de Spearman entre a satisfação no trabalho e as variáveis estudadas. Por fim, para identificar os fatores que estavam relacionados à satisfação no trabalho, foi utilizado um modelo de regressão linear. Utilizou-se o programa *Stata/* 17 SE para a análise. 

### Aspectos éticos

 Este estudo foi aprovado pelo Comitê de Ética da *Universidad Manuela Beltrán* , termo de avaliação número 20072820, e foi realizado considerando os códigos de Nuremberg e Belmont e a Resolução 8.430 de 1993, seção número 2, do Ministério de Proteção Social e Saúde da Colômbia. Foi solicitado um consentimento informado virtual antes do preenchimento da pesquisa que esteve disponível no formulário *Google* . 

## Resultados

 Entre outubro de 2021 e abril de 2022 participaram 1.221 enfermeiros, foram excluídos dois aposentados e quatro estudantes de enfermagem, restando uma amostra final de 1.215 participantes. Quanto às características sociodemográficas, a amostra foi composta majoritariamente por mulheres (79,2%), com idade mediana de 38 anos, de nível socioeconômico médio (85,8%), casadas ou vivendo em casal (51,2%) sendo graduandos/graduados (53,7%) ( Tabela 1 ). Além disso, dependendo do país de origem dos entrevistados, foram encontradas diferenças estatisticamente significativas em cada uma das variáveis mencionadas: a idade mediana mais alta foi no Equador; a menor proporção de mulheres, na Argentina; a melhor condição socioeconômica foi a da Colômbia e a maior proporção de enfermeiros com mestrado e doutoramento encontrou-se no Equador. 

 Quanto às condições de trabalho, constatou-se que a amostra era composta, em sua maioria, por profissionais de enfermagem que atuam em instituições de saúde como hospitais, unidades básicas de saúde e atenção domiciliar (73,1%), e que mais da metade estava vinculada às empresas onde prestavam os seus serviços com contrato por tempo indeterminado (58,1%). Desta amostra, cerca de metade tinha trabalho diurno ou misto (53,6% e 41,7% respetivamente); a mediana de horas trabalhadas foi de 42 e a mediana de experiência profissional foi de 10 anos ( Tabela 1 ). 

 Foram encontradas diferenças importantes nas condições de trabalho entre países. Por exemplo, na Argentina predominou a participação de enfermeiros assistenciais com maior estabilidade contratual, enquanto, na Colômbia, havia mais enfermeiros que trabalhavam durante o dia com maior número de horas trabalhadas por semana. No Equador, por sua vez, os participantes tinham mais experiência profissional ( Tabela 1 ). 

**Tabela 1 -:** Características sociodemográficas e condições de trabalho durante a pandemia de COVID-19 em profissionais de enfermagem de alguns países da América Latina (n ^*^ = 1215). Argentina, Colômbia, México, Equador e Panamá, 2021-2022

**Características**	**Total**	**Argentina**	**Colômbia**	**Equador**	**México**	**Panamá**	**valor *p*** ^†^
	n*=1215	n*=512	n*=452	n*=122	n*=66	n*=58	
**Idade/Mediana (IIQ)** ^‡^	38 (14)	40 (15)	36 (12)	**42 (16)**	36 (16)	**35 (17)**	<0,001 ^§^
Sexo feminino n*(%) ^||^	962 (79,2)	**376 (73,4)**	376 (82,3)	**106 (86,9)**	56 (84,9)	48 (82,8)	0,001 ^¶^
Nível socioeconômico n*(%)							
Baixo	149 (12,3)	**93 (18,2)**	**26 (5,7)**	9 (7,4)	13 (19,7)	8 (13,8)	
Metade	1043 (85,8)	416 (81,3)	413 (90,4)	113 (92,6)	53 (80,3)	48 (82,8)	
Alto	23 (1,9)	3 (0,6)	18 (3,9)	0 (0)	0 (0)	2 (3,5)	<0,001 ^¶^
Estado civil n*(%) ^||^							
Solteiro	464 (38,2)	199 (38,9)	171 (37,4)	41 (33,6)	30 (45,5)	23 (39,7)	
Casado ou vivendo em casal	622 (51,2)	249 (48,6)	250 (54,7)	64 (52,5)	28 (42,4)	31 (53,5)	
Divorciado ou viúvo	129 (10,6)	64 (12,5)	36 (7,9)	17 (13,9)	8 (12,1)	4 (6,9)	0,165 ^¶​^
Nível de escolaridade n*(%) ^||^							
Graduando-Graduado	652 (53,7)	373 (72,9)	175 (38,3)	24 (19,7)	52 (78,8)	28 (48,3)	
Especialização	268 (22,1)	101 (19,7)	145 (31,7)	4 (3,3)	4 (6.1)	14 (24,1)	
Mestrado	269 (22.1)	**36 (7)**	124 (27,1)	**86 (70,5)**	7 (10,6)	16 (27,6)	
Doutorado	26 (2.1)	2 (0,4)	13 (2,8)	8 (6,6)	3 (4,6)	0 (0)	<0,001 ^¶^
Tipo de instituição n*(%) ^||^							
Assistência	888 (73,1)	**466 (91)**	**256 (56)**	56 (45,9)	57 (86,4)	53 (91,4)	
Educacional, pesquisa	227 (18,7)	32 (6,3)	129 (28,2)	61 (50)	3 (4,6)	2 (3,5)	
Gestão pública, administração, comercial	100 (8,2)	14 (2,7)	72 (15,8)	5 (4.1)	6 (9,1)	3 (5.2)	<0,001 ^¶^
Tipo de contrato n*(%) ^||^							
Prazo indefinido	702 (58,1)	**357 (70,3)**	**197 (43,1)**	63 (52,5)	36 (54,6)	49 (84,5)	
Prazo fixo	286 (23,7)	87 (17,1)	147 (32,2)	30 (25)	16 (24,2)	6 (10,3)	
Outro tipo de contrato	221 (18,3)	64 (12,6)	113 (24,7)	27 (22,5)	14 (21,2)	3 (5.2)	<0,001 ^¶^
Jornada n*(%) ^||^							
Jornada diurna	651 (53,6)	**216 (42,2)**	**301 (65,9)**	76 (62,3)	40 (60,6)	18 (31)	
Jornada mista (dia/noite)	507 (41,7)	266 (52)	137 (30)	45 (36,9)	19 (28,8)	40 (69)	
Turno da noite	57 (4,7)	30 (5,9)	19 (4.2)	1 (0,8)	7 (10,6)	0 (0)	<0,001 ^¶^
**Horas de trabalho/semana mediana (RI)** ^‡^	42 (10,5)	40 (13)	**48 (6)**	40 (0)	40 (15,5)	40 (12)	0,0001 ^§^
**Experiência profissional média (RI)** ^‡^	10 (14)	10 (13)	10 (11)	**17 (17)**	10 (16)	**7,5 (12)**	0,0001 ^§^
Recomenda o trabalho n*(%) ^||^							
Sim	42 (10,5)	40 (13)	**48 (6)**	40 (0)	40 (15,5)	40 (12)	
Não	1099 (90,59)	444 (86,7)	428 (93,7)	119 (97,5)	58 (87,9)	50 (86,2)	<0,001 ^¶^

Nota: Os resultados em negrito marcam valores residuais ajustados mais extremos, em relação aos valores esperados, quando o teste de independência foi significativo na análise *post hoc;*

*n Tamanho da amostra

^†^ p Valor *de p* para nível de significância de 5%

^‡^ IIQ Intervalo interquartil

^§^ Teste Qui-quadrado de Pearson

^||^(%) Porcentagem

^¶^ Teste Kruskal-Wallis/diferença de medianas

 A Tabela 2 apresenta os resultados da satisfação no trabalho para cada dimensão e item de acordo com o país. Observa-se aí que as médias de satisfação global foram menores na Argentina e no Panamá. Existem também evidências, no nível geral, de menor satisfação nas dimensões de tensão e pressão relacionadas com o trabalho e a monotonia laboral. As dimensões das relações interpessoais com chefes e colegas foram avaliadas com maior satisfação no trabalho. 

 Ao analisar os dados, observou-se menor satisfação nos itens relacionados à fadiga/cansaço do trabalhador, carga de trabalho, falta de tempo e independência e monotonia. Pelo contrário, a melhor avaliação foi obtida nas capacidades de promoção profissional e no relacionamento entre chefes e colegas. No geral, a satisfação foi avaliada com pontuação média de 81,5 de 120 pontos, com desvio-padrão (DP) de 11,8 pontos ( Tabela 2 ). 

**Tabela 2 -:** Escores de satisfação no trabalho durante a pandemia de COVID-19 em profissionais de enfermagem em cinco países latino-americanos (n ^*^ = 1215). Argentina, Colômbia, México, Equador e Panamá, 2021-2022

**Item**		**Total**	**Argentina**	**Colômbia**	Equador n*=122	México n*=66	Panamá n*=58	**valor *p*** ^‡^
		**Média (DP)** ^†^	**Média (DP)** ^†^	**Média (DP)** ^†^	**Média (DP)** ^†^	**Média (DP)** ^†^	**Média (DP)** ^†^	
**Dimensão 1: Satisfação no trabalho**		**3,7 (0,7)**	**3,6 (0,7)**	**3,9 (0,7)**	**3,9 (0,7)**	**3,6 (0,6)**	**3,5 (0,7)**	**<0,001**
7	No meu trabalho estou muito satisfeito.	3,9 (1)	3,8 (1,1)	4 (1)	4,5 (0,7)	4 (1)	3,3 (1,1)	<0,001
10	Tenho muito pouco interesse nas coisas que faço no meu trabalho.	3,8 (1,1)	3,7 (1,1)	4.1 (1)	3,7 (1,1)	3,6 (1,2)	3,8 (1,2)	<0,001
11	Tenho a sensação de que o que estou fazendo não vale a pena.	3,5 (1,3)	3,3 (1,3)	3,8 (1,3)	3,5 (1,3)	3,4 (1,4)	3,1 (1,4)	<0,001
16	Estou convencido de que o cargo que ocupo me corresponde pela minha capacidade e preparação.	3,8 (1,2)	3,7 (1,2)	3,8 (1,1)	4 (1)	3,2 (1,3)	3,7 (1,2)	<0,002
**Dimensão 2: Estresse relacionado ao trabalho**		**2,8 (0,5)**	**2,7 (0,5)**	**2,9 (0,5)**	**2,9 (0,6)**	**3 (0,5)**	**2,6 (0,5)**	**<0,001**
2	Acho que tenho pouca responsabilidade no meu trabalho.	3,8 (1,1)	3,6 (1,1)	4,2 (1)	3,7 (1,2)	3,6 (1)	3,6 (1,1)	<0,001
3	No final de um dia normal de trabalho sinto-me muito cansado.	2 (1)	1,9 (1)	vinte e um)	2,4 (1,1)	2,5 (1,1)	1,7 (1)	<0,001
4	Muitas vezes me pego pensando em questões relacionadas ao meu trabalho.	2.1 (1)	1,9 (0,9)	2.1 (1)	2,5 (1)	2,6 (1,1)	2.1 (1.1)	<0,001
5	Muito raramente me forcei a usar toda a minha energia e capacidade para fazer o meu trabalho.	2,9 (1,2)	2,8 (1,2)	3,1 (1,3)	3 (1,2)	3 (1,1)	2,7 (1,3)	0,0001
6	Muito raramente o meu trabalho perturba o meu humor, a minha saúde ou o meu sono.	3.1 (1.2)	3.1 (1.2)	3 (1,2)	3,2 (1,1)	3.1 (1.2)	3 (1,2)	0,3395
**Dimensão 3: Competência profissional**		**3,3 (1)**	**3,1 (0,9)**	**3,7 (1)**	**3,3 (1,1)**	**3,3 (0,9)**	**3.2 (1)**	**<0,001**
22	Muitas vezes tenho a sensação de que não estou qualificado para o meu trabalho.	3,6 (1,2)	3,5 (1,1)	3,9 (1,2)	3,4 (1,3)	3,5 (1,2)	3,7 (1,2)	<0,001
23	Muitas vezes sinto que não tenho recursos suficientes para fazer o meu trabalho.	3,1 (1,3)	2,7 (1,2)	3,6 (1,2)	3,2 (1,2)	3 (1,2)	2,6 (1,3)	<0,001
24	Muitas vezes a competitividade ou a necessidade de estar à altura dos outros no meu trabalho me causam tensão ou estresse.	3,3 (1,2)	3 (1,2)	3,5 (1,2)	3,3 (1,2)	3,5 (1)	3,3 (1,2)	<0,001
**Dimensão 4: Pressão do trabalho**		**2,7 (1)**	**2,6 (0,9)**	**2,7 (1)**	**2,9 (0,9)**	**3 (1)**	**2,4 (0,9)**	**0,0002**
18	Muitas vezes sinto que não tenho tempo para fazer o meu trabalho.	2,5 (1,1)	2,5 (1,1)	2,6 (1,2)	2,6 (1,1)	2,8 (1,1)	2,3 (1)	0,167
20	Acho que meu trabalho é excessivo, não consigo dar conta do que tenho que fazer.	2,9 (1,1)	2,7 (1,1)	2,9 (1,2)	3,2 (1,1)	3,2 (1,2)	2,4 (1,2)	<0,001
**Dimensão 5: Promoção profissional**		**3,6 (0,7)**	**3,4 (0,7)**	**3,7 (0,7)**	**3,7 (0,7)**	**3,5 (0,6)**	**3,2 (0,7)**	**<0,001**
9	Tenho poucas oportunidades de aprender a fazer coisas novas.	3,2 (1,2)	3 (1,2)	3,6 (1,2)	3 (1,2)	3 (1,1)	2,7 (1,2)	<0,001
12	Geralmente o reconhecimento que recebo no meu trabalho é muito reconfortante.	3,4 (1,2)	3,3 (1,2)	3,4 (1,2)	3,8 (1,1)	3,4 (1,3)	2,9 (1,3)	<0,001
17	Tenho muitas habilidades de promoção profissional.	4.1 (1)	4 (1)	4,2 (1)	4,3 (0,9)	4 (1)	4.1 (1)	0,089
**Dimensão 6: Relacionamento interpessoal com chefes**		**4 (0,8)**	**3,9 (0,8)**	**4,1 (0,7)**	**4,2 (0,7)**	**3,8 (0,8)**	**3,9 (0,8)**	**<0,001**
13	A relação com meu chefe é muito cordial.	4 (1)	3,9 (1)	4,2 (0,9)	4,3 (0,9)	3,8 (1,1)	3,8 (1)	<0,001
19	Tenho certeza de que sei o que meus chefes esperam do meu trabalho.	3,9 (0,9)	3,9 (1)	4 (0,9)	4 (0,8)	3,8 (1)	4,1 (0,9)	0,1302
**Dimensão 7: Relacionamento interpessoal com colegas**		**4,2 (0,8)**	**4,1 (0,9)**	**4,3 (0,7)**	**4,2 (0,8)**	**3,9 (1,1)**	**4,1 (0,7)**	**0,0011**
14	A relação com meus colegas é muito cordial.	4,2 (0,8)	4,1 (0,9)	4,3 (0,7)	4,2 (0,8)	3,9 (1,1)	4,1 (0,7)	0,011
**Dimensão 8: Características extrínsecas do trabalho**		**3 (0,8)**	**2,8 (0,8)**	**3,2 (0,9)**	**3,2 (0,7)**	**2,9 (0,6)**	**2,8 (0,7)**	**<0,001**
8	Para organizar o trabalho que realizo, de acordo com meu cargo ou categoria profissional, tenho pouca independência.	2,9 (1,1)	2,8 (1,1)	3.1 (1.1)	2,8 (1,2)	2,8 (1)	2,8 (1,2)	0,003
15	O salário que recebo é adequado.	3 (1,3)	2,7 (1,2)	3,2 (1,3)	3,7 (1,1)	3 (1,3)	2,9 (1,2)	<0,001
**Dimensão 9: Monotonia do trabalho**		**3,1 (0,9)**	**2,9 (0,8)**	**3,3 (0,9)**	**3 (1)**	**3,1 (0,9)**	**2,8 (1)**	**<0,001**
1	Meu trabalho é o mesmo todos os dias, nunca muda	2,8 (1,2)	2,8 (1,1)	2,9 (1,3)	2,7 (1,3)	2,6 (1,2)	2,4 (1,3)	0,0229
21	Os problemas pessoais dos meus colegas de trabalho geralmente me afetam.	3,3 (1,2)	3 (1,1)	3,6 (1,1)	3,3 (1,1)	3,6 (1,2)	3,3 (1,1)	<0,001
**Pontuação geral**		**81,7 (11,8)**	**77,7 (10,1)**	**85,9 (11,7)**	**85,1 (13,4)**	**81,5 (10,6)**	**76,2 (9,8)**	**<0,001**

Nota: Os escores para cada dimensão e a pontuação geral estão destacadas em negrito.

*n Tamanho da amostra

^†^ (DP) Desvio-padrão

^‡^ p valor de p para nível de significância de 5%, obtido pelo teste ANOVA

**Tabela 3 -:** Características do trabalho relacionadas à pandemia de COVID-19 em trabalhadores de enfermagem em alguns países da América Latina (n ^*^ = 1215). Argentina, Colômbia, México, Equador e Panamá, 2021-2022

**Características**	**Total**	**Argentina**	**Colômbia**	**Equador**	**México**	**Panamá**	**valor *p*** ^†^
	n*=1215	n*=512	n*=452	n*=122	n*=66	n*=58	
	n*(%) ^‡^	n*(%) ^‡^	n*(%) ^‡^	n*(%) ^‡^	n*(%) ^‡^	n*(%) ^‡^	
Estresse no trabalho	1007 (84,3)	**454 (90,3)**	372 (82,7)	83 (70,9)	47 (71,2)	51 (87,9)	<0,001
Medo de ser exposto ao vírus	796 (67,7)	332 (67,3)	284 (64)	84 (70,6)	**51 (81)**	45 (79)	0,02
Aumento da carga de trabalho	972 (81,3)	436 (86,5)	366 (80,6)	74 (62,7)	45 (71,4)	**51 (89,5** )	<0,001
Incerteza no trabalho	587 (51,2)	239 (50,7)	199 (44,7)	71 (63,4)	41 (65,1)	**37 (66,1** )	<0,001
Redução de salário	219 (20,4)	87 (20)	83 (19,3)	20 (19,2)	16 (30,2)	13 (25)	0,367
Mudança para teletrabalho	431 (35,6)	109 (21,3)	**240 (53,1** )	**68 (55,7)**	10 (15,2)	4 (6,9)	<0,001
Compra de EPIs pagando do próprio bolso	645 (54,8)	254 (50,2)	206 (47,4)	86 (76,1)	**53 (80,3)**	**46 (79,3)**	<0,001

*n Tamanho da amostra

^†^p Valor *de p* para nível de significância de 5%, obtido pelo teste Exato de Fisher

^‡^ (%) Porcentagem

Em relação aos fatores de estresse no trabalho durante a pandemia de COVID-19, a maioria dos entrevistados relatou estresse (84,3%) e aumento da carga de trabalho (81,3%); pelo contrário, apenas 20,4% sofreram redução salarial e 35% reportaram mudança para o teletrabalho. Na comparação entre os países, foram encontradas diferenças importantes: na Argentina foi relatada maior proporção de estresse no trabalho (90,3%; p<0,001), enquanto a maioria dos profissionais mexicanos expressou medo devido à exposição ao vírus (81%; p=0,02); por sua vez, os enfermeiros do Panamá perceberam mais sobrecarga (89,5%; p<0,001) e incerteza no trabalho (66,1%; p<0,001); os profissionais colombianos e equatorianos foram os que mais enfrentaram a mudança para o teletrabalho (53,1% e 55,7% respectivamente; p<0,001).

Por fim, foi realizado um modelo de regressão linear, encontrando fatores socioeconômicos e de trabalho relacionados ao nível de satisfação profissional dos profissionais de enfermagem, sendo eles: nível socioeconômico, estado civil, tipo de instituição onde trabalhavam no momento da pesquisa, recomendar ou não o trabalho, estresse percebido, redução salarial, compra de EPIs pagando do próprio bolso e aumento da carga horária devido à pandemia.

 Ter um nível socioeconômico médio aumentou a satisfação em média em 3,5 pontos (IC 95%: 1,4; 5,6), enquanto estar em um nível alto aumentou a satisfação em 9,9 pontos (IC 95%: 4,3;15) quando comparado com enfermeiros de baixo nível econômico (p < 0,001). Da mesma forma, os profissionais que recomendaram seu trabalho apresentaram média 8,7 pontos (IC 95%:6,4;11) acima daqueles que não o recomendaram. Pelo contrário, os níveis de satisfação no trabalho foram reduzidos em média em 2,3 pontos (IC 95%: -3,8;-0,8) naqueles que alternam entre turnos diurnos e noturnos e em 3,8 pontos (IC 95%: -5,7;-1,9) naqueles que atuam em instituições de saúde, comparando esses dados com instituições de ensino ou pesquisa. Da mesma forma, devido à pandemia, a satisfação caiu 5,4 pontos (IC 95%:-7,3;-3,5) em quem tinha maior estresse no trabalho, 4 pontos (IC 95%:-5,7;-2,3) em quem viu seu salário reduzido, 3,2 pontos (IC 95%:-4,5;-0,8) naqueles que tiveram que comprar EPIs pagando do próprio bolso e 2,7 pontos (IC 95%:-4,6;-0,9) nos que relataram aumento na carga de trabalho ( Figura 1 ). 

**Figura 1 -:** Fatores relacionados aos escores de satisfação no trabalho durante a pandemia de COVID-19 em enfermeiros de alguns países da América Latina: Argentina, Colômbia, México, Equador e Panamá, 2021-2022

Nota: As barras cinzentas indicam a redução (valores negativos) do índice de satisfação total e as barras azuis o aumento do índice de satisfação profissional total (valores positivos) estimado pelo coeficiente β e obtido por modelo de regressão linear. Os intervalos de confiança de 95% são representados pelas linhas acima das barras; regressão com uma amostra de 1.034 participantes, r2=0,22. *Valor p <0,001 para nível de significância de 5%; ^†^ Valor de p <0,01 para nível de significância de 5%

## Discussão

 Neste estudo, observou-se um nível moderado de satisfação profissional entre os profissionais de enfermagem, com diferenças entre os países participantes. Também foi encontrado um índice de satisfação mais elevado para a Colômbia em comparação com o relatado em 2014 por 105 enfermeiros assistenciais da cidade de Bogotá, Colômbia (69,98 DP: 5,67) com a utilização do mesmo instrumento *Font-Roja* ^(^ 17 ^)^ . Pelo contrário, no México, em 2012, foi relatada uma média mais elevada de satisfação no trabalho (101; DP:10) com base nos resultados de 594 enfermeiros de quatro instituições de saúde ^(^ 18 ^)^ . Por outro lado, em Córdoba, Argentina, evidenciou-se que, de 333 inquéritos realizadas em hospitais públicos, o maior índice de insatisfação esteve relacionado ao crescimento profissional na instituição e ao valor da remuneração ^(^ 19 ^)^ . 

Os itens em que se registrou maior insatisfação (tanto intrínseca quanto extrínseca) foram aqueles que de alguma forma avaliaram como os trabalhadores perceberam suas possibilidades de crescimento, tanto na instituição quanto economicamente. Assim, uma proporção significativa dos profissionais pesquisados enfrentou suas tarefas diárias com a percepção de que não recebe um salário adequado nem teve possibilidade de promoção no local onde presta serviço.

Estes resultados díspares podem ser explicados pelo contexto da pandemia, bem como pelo tipo de instituições a que os participantes estão vinculados. Exemplo disso é que no nosso estudo constatou-se que trabalhar na área da saúde estava associado a uma menor satisfação em comparação com outras áreas.

 Da mesma forma, neste estudo as mulheres participaram em maior proporção, embora não tenham sido encontradas diferenças na satisfação por sexo. Contrariamente a isto, observou-se que as enfermeiras têm tido menor satisfação profissional em comparação com os homens ^(^ 20 ^)^ e maior estresse laboral ^(^ 21 ^)^ . Os processos de formação e emprego em enfermagem têm sido predominantemente femininos, uma vez que o ato de cuidar está socialmente vinculado às mulheres ^(^ 22 ^)^ , também de acordo com o relatório da Organização Pan-Americana da Saúde (OPAS) para a Região das Américas, aproximadamente 87% do pessoal de enfermagem são mulheres ^(^ 10 ^)^ . Isto significa que as mulheres são a maioria na força de trabalho dos cuidados e, consequentemente, têm uma maior carga por conta desses cuidados ^(^ 23 ^)^ . 

 Outro achado de interesse foi a relação entre o nível socioeconômico e a satisfação no trabalho. Esse fenômeno já foi descrito anteriormente considerando o salário e sua relação com a satisfação ^(^ 24 ^)^ , constatando que quanto melhor o nível econômico, melhor a percepção de satisfação. Além do nível socioeconômico obtido por meio do salário, é importante levar em consideração outros aspectos na avaliação da satisfação no trabalho, como longas jornadas de trabalho e necessidades de tempo e descanso ^(^ 25 ^)^ . Além disso, observou-se que as enfermeiras perceberam uma injustiça salarial, quando comparados os seus salários com o que ganhavam outras profissões com menor responsabilidade ou carga horária, disparidade salarial que pode ser determinada pela discriminação de gênero, causando insatisfação profissional em uma profissão com predominância do sexo feminino ^(^ 26 ^)^ . 

 Ressalta-se que, nos âmbitos de assistência à saúde, a pandemia causou maior impacto no trabalho, devido a jornadas prolongadas, falta ou perda de pessoal, risco de infecção e limitações em equipamentos de proteção individual ^(^ 2 ^)^ . Por exemplo, em Israel, a satisfação profissional dos enfermeiros foi avaliada durante a pandemia, sendo menor naqueles que trabalhavam em hospitais, que testaram positivo para COVID-19 ou que tinham equipamento de proteção individual deficiente ^(^ 7 ^)^ . Além disso, há maior insatisfação quando os enfermeiros não conseguem prestar os cuidados necessários aos pacientes, quando não têm apoio e supervisão, quando há falta de pessoal ou quando se sentem incapazes de fazer uma pausa e precisam fazer horas extras ^(^ 6 ^)^ . Embora os enfermeiros docentes tenham relatado uma satisfação média mais elevada do que os enfermeiros assistenciais, isso não significa que não tenham enfrentado múltiplos desafios de trabalho, como a necessidade de adaptação abrupta às tecnologias educacionais remotas. 

 Outro aspecto importante a ser analisado é a carga de trabalho, considerada o estressor que mais frequente e negativamente se relaciona com a satisfação no trabalho e a qualidade do cuidado percebida ^(^ 27 ^)^ . Por exemplo, quem trabalhava em Unidades de Terapia Intensiva teve maior exposição a estressores ^(^ 28 ^)^ e aumento na frequência de sintomas de ansiedade, depressão e estresse pós-traumático ^(^ 29 ^)^ . 

 O vínculo empregatício também desempenha um papel fundamental no bem-estar do trabalhador. No presente estudo constatou-se que apenas 58,1% dos trabalhadores de enfermagem participantes possuíam contrato por tempo indeterminado. Embora as condições de trabalho anteriores à pandemia indicassem a necessidade de reduzir a informalidade, os salários abaixo do mínimo legal ou a falta de segurança social, é possível que, com a pandemia, estes fenómenos tenham se agravado ^(^ 9 ^)^ . Por isso, os contratos estáveis devem ser favorecidos, pois têm demonstrado efeito positivo nas atitudes em relação à comunicação, inteligência emocional e empatia ^(^ 30 ^)^ , além de favorecer a saúde do trabalhador em aspectos como nível de estresse, qualidade do sono, *burnout* , taxas de rotatividade do pessoal, e otimização do atendimento ^(^ 31 ^-^ 33 ^)^ . 

 Além do exposto, a pandemia também provocou alterações na remuneração salarial em cerca de 30% dos enfermeiros que participaram do estudo. Considerou-se que a percepção de um salário baixo também foi um fator estressante para a equipe de enfermagem durante a pandemia, principalmente quando aumentaram a carga de trabalho, o cansaço e o risco de infecção ^(^ 34 ^)^ . Ainda assim, no Líbano, 38,3% dos enfermeiros que trabalharam durante a pandemia o fizeram porque precisavam de um salário para as suas famílias, mesmo que quisessem pedir demissão ^(^ 35 ^)^ . 

 Por outro lado, a pandemia de COVID-19 obrigou a uma mudança na dinâmica de trabalho, sendo relatada menor satisfação laboral por trabalhadores de turnos diurnos e noturnos, bem como um aumento da carga de trabalho, maior estresse no trabalho e incerteza laboral, com diferenças importantes entre os países participantes. Além disso, a satisfação foi menor nos itens relacionados à carga de trabalho, cansaço e falta de tempo e independência. Da mesma forma, no México, os enfermeiros que trabalharam mais de 12 horas por dia durante a pandemia sofreram maior estresse ^(^ 36 ^)^ , e no Uruguai 52,4% dos entrevistados trabalharam até 12 horas, 96,1% viram seu ritmo de trabalho acelerado e 87% sentiram um aumento na carga de trabalho quando a pandemia começou, em comparação com a que tinham normalmente ^(^ 37 ^)^ . Da mesma forma, no Peru, 38,8% dos enfermeiros relataram estresse moderado durante a pandemia ^(^ 38 ^)^ e no Equador, mais de 90% dos médicos e enfermeiros relataram esgotamento profissional ^(^ 39 ^)^ . O medo de ter que se expor ao vírus foi outra das preocupações laborais dos participantes (65,4%). Esse medo surge principalmente na prestação de cuidados diretos, o que denota vulnerabilidade a um cenário de maior carga horária e instabilidade laboral ^(^ 40 ^)^ . 

Todos esses achados mostram a necessidade de os países da América Latina estabelecerem políticas que garantam condições de trabalho mais justas, sem disparidades entre as diferentes áreas de atuação dos enfermeiros, como é o caso da Colômbia, onde foi relatada uma mediana de trabalho de 48 horas por semana, muito superior aos outros países participantes.

 Além disso, este estudo fornece evidências de como alguns aspectos sociodemográficos e trabalhistas afetam a percepção da satisfação profissional dos profissionais de enfermagem, principalmente em países onde persiste a fragilidade laboral devido às condições de contratação, à sobrecarga e ao clima organizacional, além da falta de preparo dos sistemas de saúde ^(^ 41 ^)^ . 

Embora tenha sido difícil conseguir a participação dos profissionais de enfermagem nos diferentes países latino-americanos dada a dinâmica da pandemia e as limitações de disponibilidade de tempo, as associações de enfermagem foram uma forma facilitadora para a coleta de dados. Espera-se que, em estudos futuros, seja avaliada a percepção de satisfação e sua relação com a qualidade de vida ou saúde do trabalhador, ou em situações como o trabalho múltiplo ou o desempenho simultâneo de funções, aspectos não avaliados neste estudo.

## Conclusão

A satisfação profissional da enfermagem em alguns países latino-americanos durante a pandemia diminuiu devido à percepção de pressão e tensão, falta de independência e monotonia. Além disso, os profissionais de enfermagem estavam mais satisfeitos com seu trabalho quando possuíam nível socioeconômico mais elevado e quando seu estado civil era solteiro, casado ou vivia em casal.

Adicionalmente, em termos de condições de trabalho, a satisfação foi menor naqueles que trabalhavam em ambientes de saúde, em turnos mistos (diurno e noturno) ou não recomendavam o seu trabalho. Diante das implicações que a pandemia teve no trabalho, a satisfação diminuiu para quem percebeu aumento da carga horária, maior estresse e redução salarial. O dito anteriormente confirma que, com efeito, a pandemia de COVID-19 teve um impacto negativo na insegurança no trabalho e, portanto, na satisfação profissional dos enfermeiros em alguns países latino-americanos.

Proteger a segurança do emprego dos enfermeiros é fundamental. São necessárias normas internacionais e nacionais que possam ser aplicadas juntamente com políticas laborais, para melhorar as condições de emprego dos enfermeiros e o equilíbrio entre vida pessoal e profissional. A negociação coletiva por meio da participação em sindicatos e redes pode ser uma forma de exigir emprego e condições de trabalho mais justas.
